# Detection of coronaviruses in insectivorous bats of Fore-Caucasus, 2021

**DOI:** 10.1038/s41598-023-29099-6

**Published:** 2023-02-09

**Authors:** Igor V. Popov, Olesia V. Ohlopkova, Irina M. Donnik, Petr V. Zolotukhin, Alexander Umanets, Sergey N. Golovin, Aleksey V. Malinovkin, Anna A. Belanova, Pavel V. Lipilkin, Tatyana A. Lipilkina, Ilya V. Popov, Alexandr K. Logvinov, Nikita A. Dubovitsky, Kristina A. Stolbunova, Ivan A. Sobolev, Alexander Yu. Alekseev, Alexander M. Shestopalov, Valentina N. Burkova, Michael L. Chikindas, Koen Venema, Alexey M. Ermakov

**Affiliations:** 1 Centre for Healthy Eating and Food Innovation, Maastricht University-Campus Venlo, 5900 AA Venlo, The Netherlands; 2 Agrobiotechnology Center, Faculty “Bioengineering and Veterinary Medicine”, Don State Technical University, Rostov-On-Don, 344000 Russia; 3 State Research Center of Virology and Biotechnology “Vector”, Rospotrebnadzor, World-Class Genomic Research Center for Biological Safety and Technological Independence, Federal Scientific and Technical Program On the Development of Genetic Technologies, Koltsovo, 630559 Russia; 4 Ural State Agrarian University, Ekaterinburg, 620075 Russia; 5 Southern Federal University, Rostov-On-Don, 344090 Russia; 6 Maastricht University, Youth, Food and Health, 5900 AA Venlo, The Netherlands; 7 Research Institute of Virology, Federal State Budgetary Scientific Institution “Federal Research Center for Fundamental and Translational Medicine”, 630117 Novosibirsk, Russia; 8 State Research Center of Virology and Biotechnology “Vector”, Rospotrebnadzor, 630559 Koltsovo, Russia; 9 Institute of Ethnology and Anthropology, Russian Academy of Sciences, Moscow, 119991 Russia; 10 National Research University Higher School of Economics, Moscow, 101000 Russia; 11 Health Promoting Naturals Laboratory, School of Environmental and Biological Sciences, Rutgers State University, New Brunswick, NJ 08901 USA; 12 Department of General Hygiene, I.M. Sechenov First Moscow State Medical University, Moscow, 119991 Russia

**Keywords:** Viral epidemiology, Microbiology, Zoology

## Abstract

Coronaviruses (CoVs) pose a huge threat to public health as emerging viruses. Bat-borne CoVs are especially unpredictable in their evolution due to some unique features of bat physiology boosting the rate of mutations in CoVs, which is already high by itself compared to other viruses. Among bats, a meta-analysis of overall CoVs epizootiology identified a nucleic acid observed prevalence of 9.8% (95% CI 8.7–10.9%). The main objectives of our study were to conduct a qPCR screening of CoVs’ prevalence in the insectivorous bat population of Fore-Caucasus and perform their characterization based on the metagenomic NGS of samples with detected CoV RNA. According to the qPCR screening, CoV RNA was detected in 5 samples, resulting in a 3.33% (95% CI 1.1–7.6%) prevalence of CoVs in bats from these studied locations. BetaCoVs reads were identified in raw metagenomic NGS data, however, detailed characterization was not possible due to relatively low RNA concentration in samples. Our results correspond to other studies, although a lower prevalence in qPCR studies was observed compared to other regions and countries. Further studies should require deeper metagenomic NGS investigation, as a supplementary method, which will allow detailed CoV characterization.

## Introduction

Coronaviruses (CoVs) have a high potential to adapt and evolve due to their fast replication rate, recombination in their RNAs and subsequent mutations^1^. It is hypothesized that this quality allows CoVs to overcome the interspecies barrier and adapt to new hosts^2^. In some cases, interspecies jumps could be a threat to public health and result in epidemic outbreaks of CoV infections in the human population. In recent years, we have witnessed outbreaks (MERS and SARS) and a pandemic (COVID-19) caused by animal-derived CoVs^3^. Animals such as bats, rodents, civets, cattle, swine, and camelids are recognized as hosts of CoVs diversity that have been involved in spillover and adaptation to new hosts including humans in the past, suggesting that favorable conditions may see the emergence of additional human CoVs^4^.

It has been suggested that the human CoVs HCoV-229E, HCoV-NL63, SARS-CoV, MERS-CoV, and SARS-CoV-2 may have emerged from progenitor CoVs present in bats^5^. Bats are not only hosts for CoVs, but also other emerging viruses such as Nipah, Hendra, Marburg, and Ebola viruses^6^. Several mechanisms are making these animals ideal "incubators" for emerging viruses. Limited antiviral immune reactions^7^ and inflammatory response in bats contribute to relatively long persistence of viruses, which gives emerging CoVs more time and space to get mutations for interspecies transmissions^8^. In addition to the unique antiviral immune response of bats, the ability to fly also contributes to developing new emerging viruses. Reactive oxygen species, formed as a result of active metabolism during the flight of these mammals, damage cell DNA and enzymes involved in viral replication, such as RNA-dependent RNA polymerase of CoVs^9,10^. Our previous study reveals pro-mutagenic properties of lactic acid producing bacteria within the gut microbiota of bats, which also can promote mutations in viruses of these animals^11^.

All the points mentioned above make bats the most important animals to be screened for the persistence of emerging viruses, especially CoVs. Before the COVID-19 outbreak, several papers were published on the detection of CoV in bats in Italy^12,13^, Germany^14^, France^15^, Spain^16^, United Kingdom^17^, Slovenia^18^, Netherlands^19^, Luxembourg^20^, Denmark^21^, Korea^22^, and China^23,24^. Based on this data, data from SARS-CoV and MERS-CoV outbreaks, and data on the ability of interspecies transmission of CoV, some scientists suggested that there would be a new CoV outbreak from bats^25–28^, which in the end indeed resulted in the COVID-19 pandemic. From today’s perspective, it is especially essential to conduct studies on the detection and characterization of CoVs in bats, as any obtained data can help predict the next CoV outbreaks. In this pilot study, we report the first data on qPCR screening of bats from several Fore-Caucasus regions of Russia for the presence of CoVs. We also conducted an in silico study to test the sensitivity and specificity of the pan-coronavirus assay used in this study and performed NGS of samples with detected CoV RNA.

## Results

### RNA concentration

The mean RNA concentration in liquid oropharyngeal probe samples was 2.67 ng/μL (2.58–2.77 ng/μL) and in liquid rectal samples was 2.71 ng/μL (2.59–2.82 ng/μL) There are no significant differences in RNA concentration in oropharyngeal and rectal swabs (p = 0.997) (Supplementary materials, File S1, Fig. S1).

### qPCR screening

Positive signals on detection of CoV RNA were found in five samples from five different animals: samples from two Kuhl's pipistrelles, two serotine bats, and one lesser horseshoe bat. CoV RNA was detected in four oropharyngeal swab samples and one rectal swab sample. A detailed database of bats and CoV detection can be found in supplementary materials, File S2.

**Figure 1:**
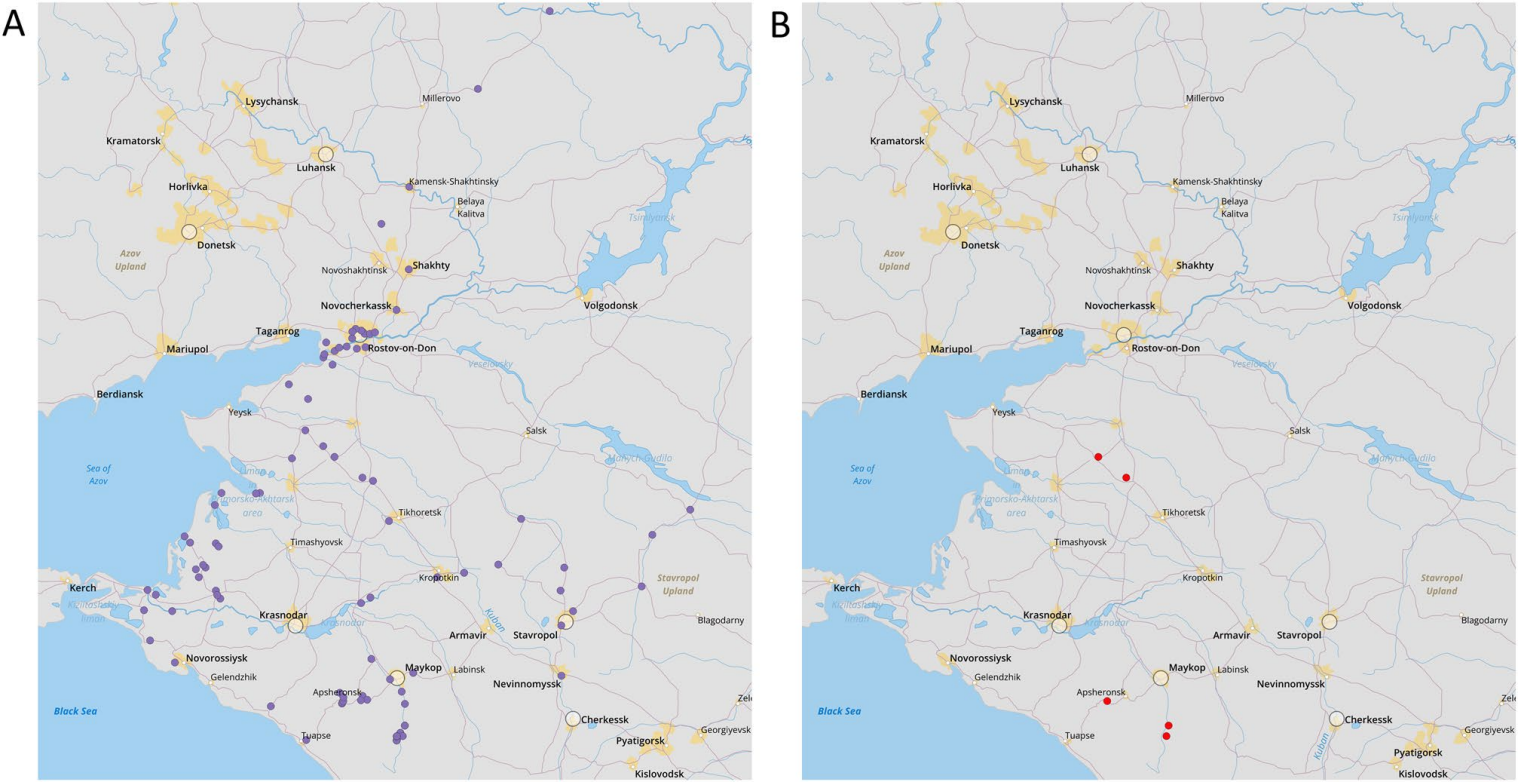
Global positioning system (GPS) showing (**A**) probe locations in this study, and (**B**) locations, from where probes with detected CoV RNA were taken. (This map was created by QGIS version 3.28, which can be accessed on https://qgis.org/en/site/).

The mean proportion of detected CoVs in the studied population of bats is 3.33% (CI 1.1–7.6%). There is no significant effect of bat species (χ^2^ = 6.23, df = 6; p = 0.39), swab origin (χ^2^ = 1.83, df = 1; p = 0.18), sex (χ^2^ = 1.38, df = 1; p = 0.24), and sample collection site (χ^2^ = 2.9, df = 3; p = 0.4) on the detection of CoV RNA, likely also due to the low number of positive samples. There are no significant predictors for CoV RNA detection observed, the results of the simple logistic regression are given in the Table 1. The global positioning system (GPS) representing the locations of collection sites is shown in Fig. 1.

**Table 1:** The proportion of detected CoV RNA in studied samples and simple logistic regression results for predictors of CoV detection. Confidence intervals are not reported for odds ratio as standard error values are extremely high due to the low number of CoV RNA detection in studied probes from 150 bats. The Clopper-Pearson interval was used to calculate confidence intervals (CI). p-value is shown for unadjusted odds ratios.

Predictor	Proportion of detected CoV RNA (CI %)	Unadjusted odds ratio	p-Value
Bat species	3.33% (1.1–7.6%)		
*Nyctalus noctula*	0	1 (reference)	
*Pipistrellus kuhlii*	9.1% (1.1–29%)	18.9	0.99
*Eptesicus serotinus*	8.3% (1–27%)	18.8	0.99
*Rhinolophus hipposideros*	3.8% (0.5–13%)	17.3	0.99
*Rhinolophus ferrumequinum*	0	0	1
*Myotis daubentonii*	0	0	1
*Barbastella barbastellus*	0	0	1
Constant (baseline odds)		− 21.2	
Swab			
Rectal		1 (reference)	
Oropharyngeal	2.7% (0.7–6.7%)	1.4	0.21
Constant (baseline odds)	0.7 (0–3.7%)	− 5	
Sex		5	
Male	3.1% (0.6–8.8%)	1 (reference)	
Female	3.8% (0.5–13%)	1	0.26
Constant (baseline odds)		− 3.9	
Region		5	
Rostov region	0	1 (reference)	
Adygea Republic	4.2% (0.9–11.7%)	18.6	0.99
Krasnodar Krai	4.5% (0.6–15.5%)	17.6	0.99
Stavropol Krai	0	0	1
Constant (baseline odds)		− 21.2

### In silico primer sensitivity and specificity

**Figure 2:**
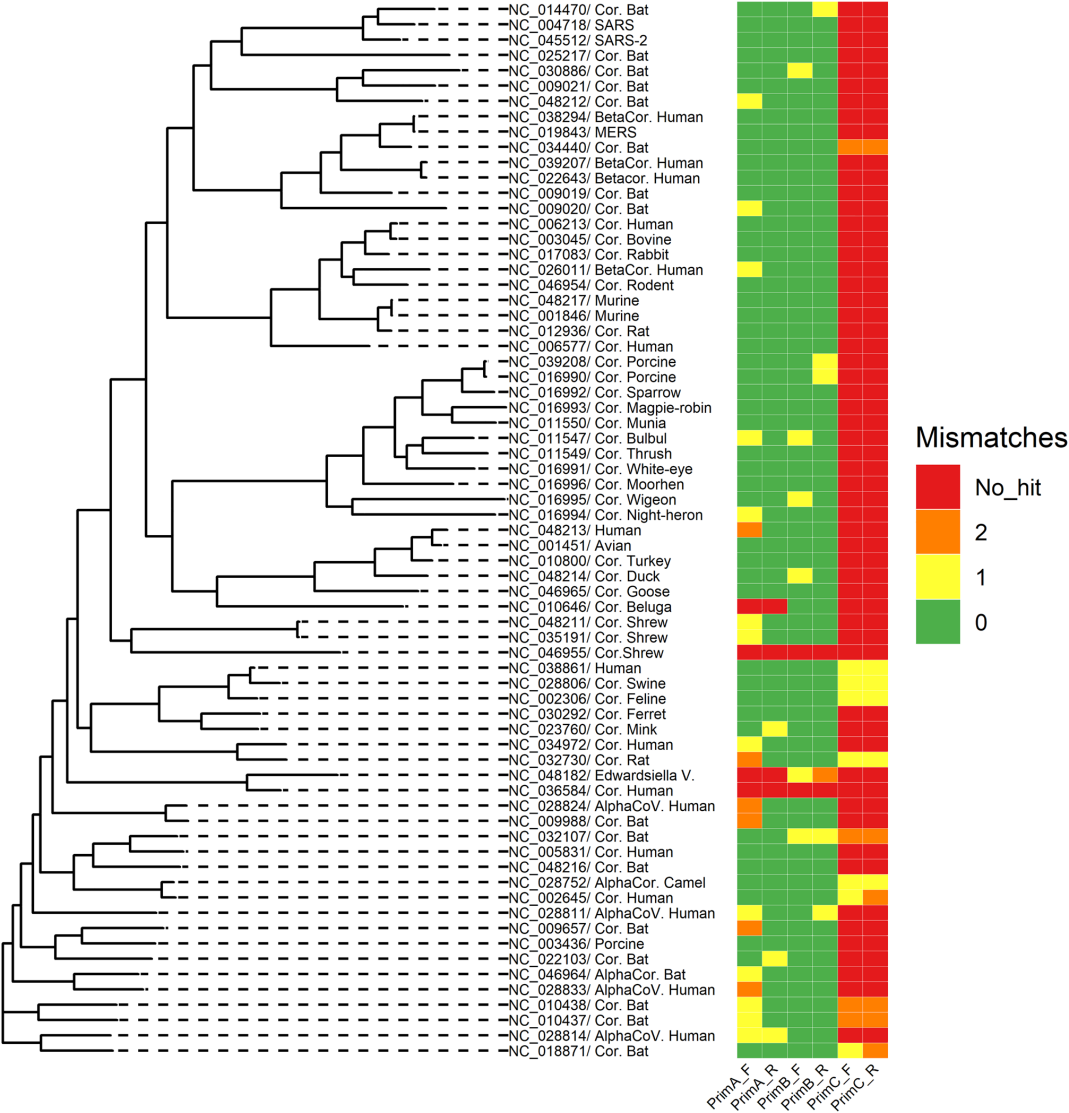
Phylogenetic tree built using 68 full reference genomes of CoV species and one reference genome of Edwardsiella virus pEt-SU (indicated by the NC-code from the NCBI RefSeq database). The rectangular heatmap represents the number of mismatches for forwards (F) and reverse (R) primers from Vijgen et al.^29^ (PrimeA), Lelli et al.^13^ (PrimeB), and Watanabe et al.^30^ (PrimeC) during in silico PCR testing.

In silico analyses were conducted on the ability of pancoronavirus primers to detect RNA of various types of CoVs. As a result, it was established that the used degenerate primers from Vijgen et al.^29^ study could detect at least 65 CoV species from available reference genomes belonging to the *Coronaviridae* family. No hits were detected against other viruses, therefore, specificity could be assumed as 100% (Fig. 2). Most mismatches were observed in the forward primer—1 mismatch with 24.18% of published CoV genomes and 2 mismatches with 6.59% of CoV genomes. There were more than two mismatches in the genomes of 3 CoVs: Beluga Whale CoV SW1, unidentified human CoV isolate FT1407-6/2014, and Shrew-CoV/Tibet2014. Pairs of primers from Lelli et al.^13^ showed similar sensitivity. However, they also detected the Edwardsiella virus pEt-SU reference genome, and based on this we cannot assume 100% specificity for this pair of primers. Primers from Watanabe et al.^30^ study mostly had more than two mismatches in used reference CoV genomes, which is above the number of allowed mismatches. Detailed results of in silico analyses are shown in the supplementary file (File S3). The sensitivity of forward and reverse primers is presented in Table 2.

**Table 2:** Rate of mismatches of forward and reverse pan-coronavirus primers (15 November 2022).

Sourse	Primer	Mismatches			
		0	1	2	NO HIT
Vijgen et al.^29^	Forward	67.65%	19.12%	8.82%	4.41%
	Reverse	91.18%	4.41%	–	4.41%
Lelli et al.^13^	Forward	89.70%	7.36%	–	2.94%
	Reverse	89.70%	7.36%	–	2.94%
Watanabe et al.^30^	Forward	–	10.30%	5.88%	83.82%
	Reverse	–	7.36%	8.82%	83.82%

### Results NGS of samples with detected CoV RNA

After performing library preparation with NEBNext ARTIC SARS-CoV-2 Library Prep Kit (New England Biolabs, England) we did not observe amplified cDNA of SARS-like CoVs’ gRNA suitable for NGS. Results of electrophoresis of cDNA amplicons can be found in supplementary materials, File S4 (Fig. S2). Although, sequencing of target genome fragments of SARS-like CoVs was not successful due to the absence of amplicons, we obtained metagenomic data from samples with detected CoV RNA. According to the taxonomical identification of raw reads (FASTQ) with Kraken2, there were 30 reads of *Coronaviridae* genomes in data obtained from MiSeq runs and 54 reads of *Coronaviridae* genome in data obtained from NovaSeq runs. Results of taxonomical identification can be found in supplementary materials, File S5. Some assembled contigs had 89–100% identity with SARS-related CoVs and HcoV-OC43 records from the NCBI Virus database with 3–16% coverage. The length of these contigs is from 458 to 1236 bp. Gene annotation by VADR was not successful, however, part of the contigs was aligned to the SARS-CoV-2 reference genome used for this tool. Assembled contigs and outcomes of BLAST search of similar viral nucleotide sequences in the NCBI Virus database (ssRNA(+) records) can be found in supplementary materials, File S6.

## Discussion

Compared to CoVs from other animals bat-borne CoVs are especially dangerous as some unique features of bat physiology boost the mutation rate in CoVs, which themselves have a high mutation rate already^4,11^. To predict future CoV outbreaks, more and improved surveillance is required to investigate CoV prevalence among bats and to characterize their genomic features. In this study, screening of CoV prevalence in bats from multiple locations in the European part of Southern Russia was performed using qPCR with pan-coronavirus primers.

The proportion of detected CoVs in the studied population of 150 bats is 3.33% (5/150; 95% CI 1.1–7.6%). In most previous studies CoV prevalence in bats is higher. Bonilla-Aldana et al. conducted a meta-analysis of 33 reports of PCR screening of bats and established that prevalence by RT-PCR in these studies (n = 14,295 bats) for CoV was 9.8% (95% CI 8.7–10.9%)^31^. However, there are some studies with a low rate of CoV RNA detection by PCR. Mendenhall et al. reported that they did not detect CoVs with PCR targeting polymerase gene in 431 pooled oral-rectal samples from six bat species and 1124 urine and faecal samples from cave nectar bat (*Eonycteris spelaea*)^32^. Although, the authors did detect CoV genomes with next-generation sequencing in pooled faecal material and pooled urine from *E. spelaea*^32^. Cappelle et al. also reported a relatively low proportion of positive PCR tests for CoVs in Cambodian bats—4.2% (24/573) in bats from Kampot, and 4.75% (22/463) in flying foxes from Kandal^33^, which overall corresponds to our results. Xu et al. detected CoV RNA in 5.3% (50/951 intestinal specimens) in bats of eight species from four provinces and the Tibet Autonomous Region of China by pan-coronavirus RT-PCR screening^34^, which is also below pooled average prevalence of CoVs in bats according to Bonilla-Aldana et al.^31^. Corman et al. conducted PCR screening of 1868 faecal, blood, and intestinal tissue specimens from 1560 bats in Costa Rica, Panama, Ecuador, and Brazil. They also used an assay targeting the RNA-dependent RNA-polymerase of CoVs and detected CoV RNA in 50 specimens from nine different bat species with overall CoV prevalence in 2.7 % of the total samples^35^. Corman et al. included more samples and animals in screening than in our study, which likely means that the smaller sample size did not have a negative impact on the overall results of PCR CoV screening. Some of the highest CoV prevalence in bats are reported in China by Ge et al.—50% (138/276 faecal samples)^36^, in Italy by Balboni et al.—42% (19/45 faeces samples)^12^, and in the Philippines by Tsuda et al.—29.6 % (53/179 faeces samples)^37^.

We detected CoVs in *P. kuhlii*, *E. serotinus*, and *R. hipposideros*. Various types of CoVs were detected in these species before. For example, Lelli et al.^13^ detected alpha- and betaCoVs in *P. kuhlii* and betaCoVs in *R. hipposideros* in Italy. In the other study, which was conducted at the same time in Italy, betaCoV was also detected in *E. serotinus*^38^. CoVs have been detected previously in Asian populations of *E. serotinus* in Kazakhstan and Korea^39,40^.

To investigate pan-coronavirus primer efficiency, we performed in silico analyses of the ability of used primers and additional pairs from other studies to detect several CoV species. Vijgen et al. designed their primers based on a 251-bp fragment of the polymerase gene of CoVs known at the time and checked their sensitivity on 14 CoVs of human and animal origin^29^. We established that these primers could detect at least 65 CoV species, including SARS-like CoV (File S3). This is because Vijgen et al. designed their primers based on a target region that is highly resistant to mutations and variations, which occurred in the most of used CoV genomes for the in silico assay. In addition, we tested pairs of primers from Lelli et al.^13^ and Watanabe et al.^30^, that are also designed for a highly conserved region of the RNA-dependent RNA polymerase gene of CoVs. In silico, primers from Lelli et al. showed similar sensitivity to primers from Vijgen et al., however, the specificity was lower, as they detected the Edwardsiella virus pEt-SU genome in addition to CoVs. The primers from Watanabe et al.^30^ mostly detected bat CoVs, but the overall sensitivity was relatively low compared to the other pairs of primers. We suggest that the first two pairs of primers showed better sensitivity, as they are degenerate primers and can cover more possible nucleotide combinations of targeted polymerase gene and naturally detect more CoVs. The study by Watanabe et al.^30^ is primarily aimed at the detection of bat CoVs, which is why they probably mostly used bat CoVs genomes for primers design. We believe that this approach has some limitations, as degenerate pairs of primers are able to detect the target region of the CoV genome with various nucleotide sequence combinations, and most importantly they are more suited for the qPCR assay, which is more efficient for the initial screening. That is why we used degenerate pairs of primers for this first epizootiological study of CoVs in the bats population of Fore-Caucasus. Our results from in silico analyses support the statement that pan-coronavirus assays can be an effective tool for the management of emerging CoV diseases^41,42^.

We did not observe a significant effect of bat species, swab origin, sex, or sample collection site on the detection of CoV RNA by χ^2^ tests and simple logistic regression, due to the low prevalence detected. Also, there were no significant differences in RNA concentration in oropharyngeal and rectal swabs, which additionally states that swab origin did not contribute to the CoV RNA detection rate. But we should note that the mean RNA concentration was probably too low for NGS analysis sufficient for the effective assembly of CoV genomes and their annotation.

The overall CoV RNA detection rate is probably relatively small for the studied sample size (150 bats) for predictor calculations. As mentioned before, Cappelle et al. established a similar rate of CoV RNA detection in samples from bats in Cambodia and Kandal. The sample size in their study is larger, and they calculated that the rate of CoV detection is significantly higher in juveniles and immature individuals than in mature adults^33^. We suggest that there are two ways to define a significant predictor for CoV RNA detection in bats population of the Fore-Caucasus. The first, is to increase the sample size with a similar amount of sample collection sites to increase the sample size effect according to the basic principles of biostatistics^43^. The second is to reduce the amount of sample collection sites with the same sample size, as in studies with a lower amount of sample collection locations, the CoV detection rate in bats is relatively higher. For example, Alkhovsky et al. collected 120 oral swabs and 77 faeces samples from bats in 6 caves and 2 house attics in the Sochi National Park of Russia and detected an overall CoV prevalence of 14%. Most interesting is that the prevalence of SARS-like CoV Khosta-1 in greater horseshoe bats from Khosta 1 cave is 62.5% (15/24 faeces samples). However, the authors did not perform any statistical testing to investigate predictors for CoV detection rate^44^. Our study included many more collection sites (Fig. 1a), as we aimed to collect samples from different parts of four studied regions. This pilot study creates a foundation for future studies of CoVs in bats in the European part of Southern Russia. For example, five collection sites where we detected bat CoVs (Fig. 1b) can be included in the next studies with the inclusion of more animals from these locations. The detection rate of CoVs in bats’ populations strongly depends on the characteristics of colonies included in the study, particularly colony size, reproduction/hibernation status of the animals, number of species in the colony, and the location of the colony. A sampling for future studies of Fore-Caucasus bats’ populations should be done considering not only the results of this study but also these features of bats’ colonies.

NGS data from samples with detected CoV RNA was not sufficient for the detailed characterization of identified CoVs with qPCR. Probably, the concentration of extracted RNA was too low to obtain good quality genomic CoVs’ data suitable for gene annotation and following phylogenetic analysis. We performed three attempts for NGS. The first one included sequencing of shotgun metagenomic libraries consisting of 2 × 150 bp cDNA fragments with NovaSeq (Illumina, USA) generating 800 million paired reads per sample. Taxonomical analysis of raw reads from this run resulted in the identification of several reads similar to the SARS-related CoV records from the NCBI RefSeq database used by Kraken2. However, we did not assemble any contigs that were precisely recognized as any CoV by BLAST or VADR. Next, we tried to perform library preparation targeting SARS-related CoVs by RT-PCR with NEBNext ARTIC SARS-CoV-2 Library Prep Kit (New England Biolabs, England). We hypothesized that the amplification of SARS-related CoVs’ genomic fragments with primer pools from this kit could improve the outcome of obtaining at least a partial genome of detected CoVs, but amplification during library preparation was not successful. Then we decided to use longer cDNA fragments (2 × 300 bp) for the third attempt at a shotgun metagenomic library preparation and sequence it with the generation of 40 million paired reads per sample. Compared to the first run there were more identified reads similar to *Coronaviridae* records in the obtained raw reads. However, this did not result in the assembly of useful contigs. We should acknowledge that according to BLAST search in NCBI Viruses database there are some records of SARS-related CoV and HCoV-OC43 that share 89–100% identity with part of assembled contigs, but they also are similar to other even non-viral records as their length does not allow for proper alignment during BLAST search. According to the taxonomical identification of raw and assembled metagenomic data, we assume that we identified betaCoVs, which are not SARS-related, as otherwise parts of the SARS-like CoVs would be amplified during library preparation targeting their specific regions.

Babiker et al. performed screening of SARS-CoV-2 and other respiratory viruses in human samples with a similar study design, including RT-PCR screening following metagenomic NGS of positive samples with MiSeq platform and similar sequencing depth (42 million reads per sample)^45^. In all cases, they detected the same viruses with metagenomic NGS as were detected with RT-PCR. The number of minimum SARS-CoV-2 reads from NGS data per one sample detected with KraenUniq was 27, which allowed Babiker et al. to assemble at least 6 genome regions from the sample with the least detected SARS-CoV-2 reads^45^. In our study, we detected reads of various CoV species (Supplementary File S5), and we suggest that the number of reads per sample from NovaSeq and MiSeq data was relatively low for the proper CoV genomes assembly. Crook et al. also performed a search of CoVs in bats, however, they only used metagenomic NGS of 2x150 cDNA fragments with MiSeq and longer cDNA fragments with Oxford nanopore GridION (Oxford Nanopore Technologies, Great Britain)^46^. The largest single contig identified as part of the CoV genome and assembled from MiSeq data was ~ 7 kb, while in our study the largest contig was ~ 1.2 kb. This also could confirm that the RNA concentration could affect the results of metagenomic NGS. However, we cannot compare the RNA concentration obtained in our work and in Crook et al. study, since the authors did not provide any relevant data on this. One of the possible ways for studying CoVs phylogenetics is amplifying long cDNA fragments with PCR following capillary sequencing, as described in Lelli et al.^13^ and Watanabe et al.^30^ studies. We should mention that this approach is also not the best option, as some CoVs can be not detected with PCR. For example, Kohl et al. reported that in their study, metagenomic NGS identified bat viruses that PCR did not detect^47^.

This study has some limitations, as we used only oropharyngeal and rectal swabs, which probably resulted in low RNA concentration and the impossibility of detected CoV characterization based on metagenomic NGS data due to this. Some other studies also include tissue, urine, faecal samples, and even exoparasites for CoV screening^32,34,35,48^. We did not collect tissue biopsies or urine from bats to prevent keeping animals in captivity for sampling. Faecal samples were not collected as it is impossible to predict bat species origin in some cases, contributing to some uncertainties during biostatistical analysis. However, future studies should include not only swab sampling, but also tissues and faeces to increase the RNA concentration needed for various sequencing studies. Also, our future studies should include not only samples of different origins, but also some other approaches, such as capillary sequencing of amplified CoV genes. Metagenomic NGS should be used as a main supplementary method to assist in characterizing previously unknown CoV genomes, that could not be done with PCR.

## Conclusions

This study provides the first data on CoVs detection, with a mean overall prevalence of 3.33% (95% CI 1.1–7.6%). Although these results correspond to similar studies conducted in other regions and countries, further studies are required to investigate the reservoir of CoVs in bats and other animals, and to be able to predict a possible next pandemic. Metagenomic NGS did not provide sufficient data for detailed CoV characterization, but in raw NGS data of all qPCR-positive samples, betaCoV reads were detected with a taxonomical identification tool, which supports the results of the qPCR screening.

## Methods

### Sampling

A total of 300 probes from 150 bats of seven species (oropharynx and rectal swabs from each animal) were collected in Rostov region, Adygea Republic, Krasnodar Krai, and Stavropol Krai from March 2021 to June 2021. The following species of bats were included in the study: common noctule (*Nyctalus noctula*), Kuhl's pipistrelle (*Pipistrellus kuhlii*), serotine bat (*Eptesicus serotinus*), lesser horseshoe bat (*Rhinolophus hipposideros*), greater horseshoe bat (*Rhinolophus ferrumequinum*), Daubenton's myotis (*Myotis daubentonii*) and western barbastelle (*Barbastella barbastellus*). Identification of bat species was performed by a trained zoologist-taxonomist in the location site of sampling. The characterization of animals included in the study is represented in Table 3.

**Table 3:** Characterization of animals, from which probe sampling for the study was done.

Variable		n	%
Bat species		150	100
* Nyctalus noctula*		43	28.7
* Pipistrellus kuhlii*		22	14.7
* Eptesicus serotinus*		24	16
* Rhinolophus hipposideros*		53	35.3
* Rhinolophus ferrumequinum*		1	0.7
* Myotis daubentonii*		1	0.7
* Barbastella barbastellus*		6	4
Sex	Bat species	150	100
Male	All	53	35.3
	*Nyctalus noctula*	18	12
	*Pipistrellus kuhlii*	10	6.7
	*Eptesicus serotinus*	7	4.7
	*Rhinolophus hipposideros*	14	9.3
	*Rhinolophus ferrumequinum*	2	1.3
	*Myotis daubentonii*	1	0.7
	*Barbastella barbastellus*	1	0.7
Female	All	97	64.7
	*Nyctalus noctula*	25	16.7
	*Pipistrellus kuhlii*	12	8
	*Eptesicus serotinus*	17	11.3
	*Rhinolophus hipposideros*	39	26
	*Rhinolophus ferrumequinum*	4	2.7
	*Myotis daubentonii*	0	0
	*Barbastella barbastellus*	0	0
Region	Bat species	150	100
Rostov region	All	24	16
	*Nyctalus noctula*	12	8
	*Pipistrellus kuhlii*	7	4.7
	*Eptesicus serotinus*	5	3.3
	*Rhinolophus hipposideros*	0	0
	*Rhinolophus ferrumequinum*	0	0
	*Myotis daubentonii*	0	0
	*Barbastella barbastellus*	0	0
Adygea Republic	All	72	48
	*Nyctalus noctula*	7	4.7
	*Pipistrellus kuhlii*	4	2.7
	*Eptesicus serotinus*	0	0
	*Rhinolophus hipposideros*	53	35.3
	*Rhinolophus ferrumequinum*	6	4
	*Myotis daubentonii*	1	0.7
	*Barbastella barbastellus*	1	0.7
Krasnodar Krai	All	44	29.3
	*Nyctalus noctula*	21	14
	*Pipistrellus kuhlii*	8	5.3
	*Eptesicus serotinus*	15	10
	*Rhinolophus hipposideros*	0	0
	*Rhinolophus ferrumequinum*	0	0
	*Myotis daubentonii*	0	0
	*Barbastella barbastellus*	0	0
Stavropol Krai	All	10	6.7
	*Nyctalus noctula*	3	2
	*Pipistrellus kuhlii*	3	2
	*Eptesicus serotinus*	4	2.7
	*Rhinolophus hipposideros*	0	0
	*Rhinolophus ferrumequinum*	0	0
	*Myotis daubentonii*	0	0
	*Barbastella barbastellus*	0	0

Sampling was performed with sterile swabs with the 2.5 mm diameter of the collecting part (Biomedical Innovations LLC, Russia). The probe was placed in a sterile 2 ml tube filled with a transport medium for virus-containing samples TVM (Biomedical Innovations LLC, Russia). After the procedure, each animal was released within the range they were caught to exclude the negative impact on the environment. Animals were not kept in captivity. Tubes were delivered to the laboratory within 24 h after sampling.

### RNA extraction

RNA extraction from liquid probe samples was carried out using the QIAamp Viral RNA mini kit (Qiagen, Germany) according to the manufacturer's protocol. Extracted RNA was divided into aliquots for quality and concentration studies using fluorimetry using the Qubit RNA HS Assay Kit (Thermo-Fisher Scientific, USA), DNase processing and reverse transcription, and the preparation of negative reverse transcription controls. Reverse transcription was performed using the MMLV RT kit (Evrogen, Russia) according to the manufacturer's protocol for liquid virus-containing samples using random decanucleotide primers.

### Primers and qPCR

For qPCR screening we used pan-coronavirus primers with sequences: Cor-FW (5′-ACWCARHTVAAYYTNAARTAYGC-3′) and Cor-RV (5′-TCRCAYTTDGGRTARTCCCA-3′) as described by Vijgen et al.^29^. The synthesis of oligonucleotides, including degenerate primers, was carried out by Evrogen. qPCR reactions were performed using hot-start PCR mixture qPCRmix-HS and SYBR Green dye (Evrogen, Russia) on a QuantStudio 5 amplifier (Thermo-Fisher Scientific, USA). Optimization of the reaction parameters was carried out according to the following parameters: serial dilution of the matrix (reaction efficiency analysis), annealing temperature, annealing time, elongation temperature, elongation time. The optimal annealing temperature was selected using gradient PCR using the capabilities of the Veriflex block of a QuantStudio 5 cycler (Thermo-Fisher Scientific, USA). The melting curve analysis was carried out in the cases of all PCR settings. The PCR program was as follows: 1 cycle of 5 min at 95 °C, 54 cycles of 15 s at 94 °C, 30 s at 44 °C, 30 s at 60 °C, 1 cycle of melting assay − 0.15 °C increment starting at 60 °C and up to 95 °C. Complete negative control qPCR reactions were performed using DEPC water as a matrix with the main reactions. The synthesized sequence corresponding to 251 target nucleotides of entries EF584908.1 (Asian leopard cat coronavirus), DQ249235.1 (Bat coronavirus HKU2), and AF304460.1 (Human coronavirus 229E) were used as the reaction/primers optimization matrix and positive controls in the subsequent reactions. The sequence was synthesized by Evrogen and diluted to 1 ng/μL to serve as the template. The cutoff values were determined automatically by QuantStudio software. The analysis of PCR data was carried out in the Design and Analysis environment (Thermo-Fisher Scientific, USA). The qPCR screening was performed in three independent replicates.

### Statistical analysis

Data for RNA concentration was not normally distributed according to the Kolmogorov–Smirnov test. To compare mean RNA concentration in probes of different swab origin unpaired U-test was performed. Pearson χ^2^ tests to explore the effect of (i) bat species, (ii) swab origin, (iii) sex, and (iv) sample collection site on the detection of CoV RNA were performed. For predictors calculations, simple logistic regression was performed. For calculation of confidence intervals for binomial proportions, the Clopper–Pearson interval was used. Data were presented as mean and 95% CI where ap-propriate. Differences were considered statistically significant when p < 0.05.

### In silico analysis of pan-coronavirus assay

The sensitivity (% of target organisms detected) and specificity (% of non-target organisms detected) of primes were tested using in silico PCR approach^49^. From the degenerate primers pairs from Vijgen et al.^29^, Lelli et al.^13^, and Watanabe et al.^30^, all possible pairs of primers without ambiguous bases were generated using a custom R script (R v4.1.0, R Foundation for Statistical Computing, Vienna, Austria). Each generated primer pair was tested against a search database with the "primersearch" function from "EMBOSS" package^50^. The maximum number of allowed mismatches was set to two per primer. Two search databases were compiled from genome sequences available in the NCBI refSeq database (15 November 2022). Available genome sequences from the *Coronaviridae* family (68 in total) were used to test sensitivity, all available virus genomes (12026 in total including the *Coronaviridae*) were used to test specificity. Whole genomes from the *Coronaviridae* family were aligned with MAFFT^51^, and the phylogenetic tree was constructed using FastTree^52^ and visualized with "ggtree" package^53^.

### NGS of samples with detected CoV RNA and bioinformatical analysis of obtained data

**Figure 3:**
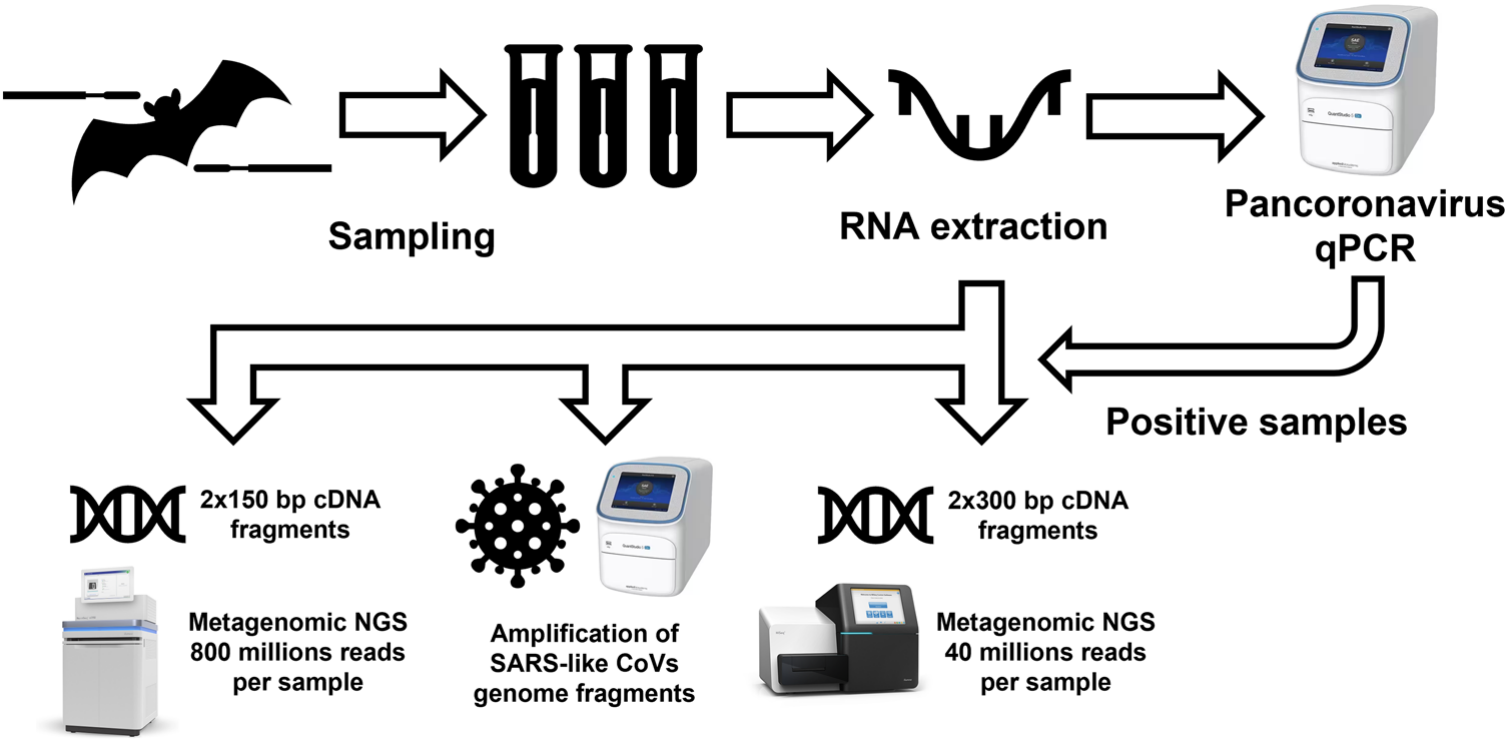
Flow-chart representing conducted molecular studies.

RNA extraction was carried out using the QIAamp Viral RNA mini kit (Qiagen, Germany) according to the manufacturer's protocol. Single-strand cDNA synthesis was performed with reverse transcriptase M-MuLV–RH (Biolabmix, Russia), and double-strand cDNA synthesis was carried out using Klenow fragment (SibEnzyme, Russia). Library preparation was performed using three different aliquots from one sample with NEBNext Ultra II DNA Library Prep Kit for Illumina (New England Biolabs, England), NEBNext ARTIC SARS-CoV-2 Library Prep Kit (New England Biolabs, England), and LIB Display kit (DNA Display, Russia) according to the manufacturers’ protocols. The concentration of cDNA in the fifth sample aliquot was not sufficient for library preparation with NEBNext Ultra II DNA Library Prep Kit (New England Biolabs, England). We used Illumina NovaSeq 6000 (Illumina, USA) for NGS with the generation of 800 million paired reads per sample of the library prepared with NEBNext Ultra II DNA Library Prep Kit and consisting of 2 × 150 bp cDNA fragments and Illumina MiSeq (Illumina, USA) for NGS with the generation of 40 million paired reads per sample of the library prepared with LIB Display kit consisting of 2 × 300 bp cDNA fragments. Graphical representation of molecular studies is shown in Fig. 3.

Quality control, filtering, and trimming of obtained FASTQ files was performed with fastp (version 0.23.2) software^54^. Taxonomical identification of raw reads was conducted with Kraken2 (version 2.1.2) using the NCBI RefSeq database as a reference^55^. Visualisation of taxonomical identification of metagenomics data was performed with Krona (version 2.8.1)^56^. SPAdes (version 3.15.4)^57^ and MEGAHIT (version 1.2.9)^58^ were used for *de novo* assembly of metagenomes. Binning of obtained contigs was performed with the abovementioned tools for taxonomical identification and seqtk tool (version 1.3)^59^. We used BLAST^60^ and NCBI Virus database^61^ for search of similar viral nucleotide sequences and VADR^62^ for validation and gene annotation of assembled contigs.

### Ethical approval

The study was approved by the ethics committee of the Don State Technical University, Rostov-on-Don, Russia (protocol number 67-43-4). Experimental procedures for this report did not include any in vivo studies. ARRIVE guidelines: not applicable^63^. The collection of probes was carried out according to the Sanitary and Epidemiological Regulations SP 3.2.1288-03. All studies were conducted according to the protocols recommended by the manufacturers of used kits, devices and software. All procedures were performed in accordance with relevant guidelines' in the manuscript.

## Supplementary Information

Supplementary Information 1.

Supplementary Information 2.

Supplementary Information 3.

Supplementary Information 4.

Supplementary Information 5.

Supplementary Information 6.

## Data Availability

All reference data used for this study are retrieved from NCBI open database and are in open access. Supplementary File S1 contains a figure representing RNA concentration in oropharynx and rectal swabs from bats. Supplementary File S2 contains a database of studied bat species, bat sex, regions where sampling was done, and CoV RNA detection rate. Supplementary File S3 contains reference names of used CoV genomes. Supplementary File S4 contains the results of gel electrophoresis of cDNA fragments prepared with ARTIC SARS-CoV-2 Library Prep Kit. Supplementary File S5 contains the results of the taxonomical identification of raw reads. Supplementary File S6 contains assembled contigs and results of BLAST search in the NCBI Virus database (ssRNA(–) records). Raw metagenomic sequencing data are available in Sequence Read Archive (SRA) under accession numbers SRR22460268, SRR22460267, SRR22460266, SRR22460265, SRR22460264, SRR22460263, SRR22460271, SRR22460270, SRR22460269.
