# Supplementary figures and images for: Detection of coronaviruses in insectivorous bats of Fore-Caucasus, 2021
Source: Sci Rep. 2023 Feb 9;13:2306. doi: 10.1038/s41598-023-29099-6 (PMC9909659; doi:10.1038/s41598-023-29099-6)

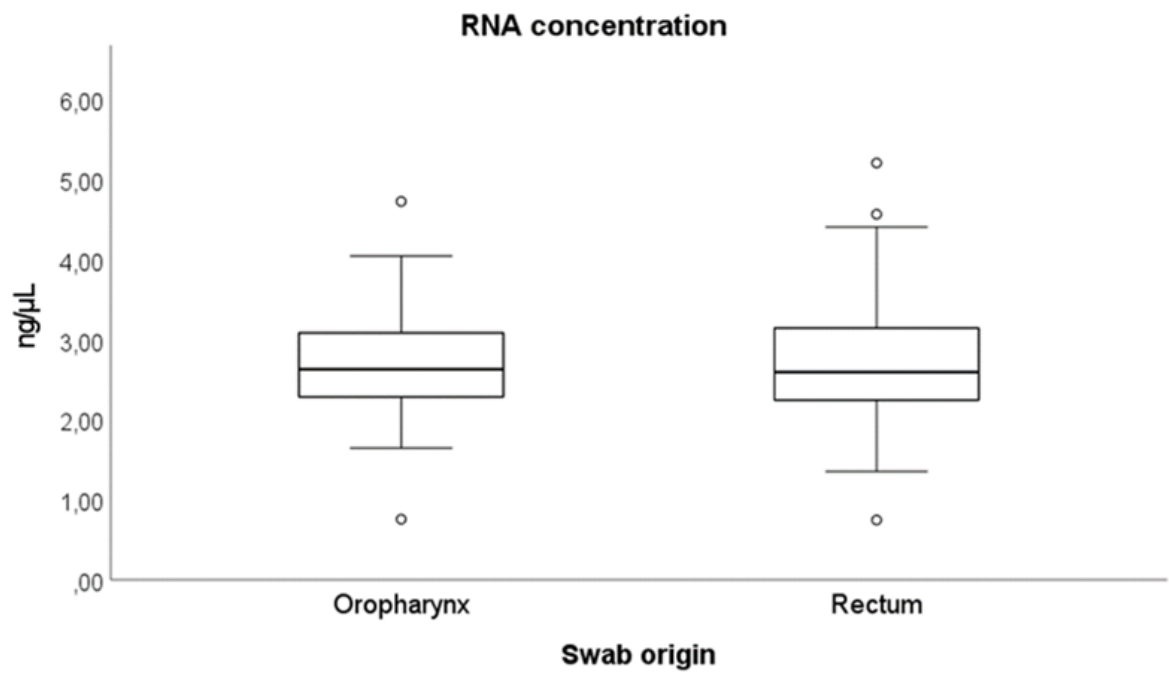

**Figure S1.** RNA concentration (ng/μL) in oropharynx and rectal swabs from bats.

Supplement: Supplementary file 1 — Supplementary Information 1. [file 41598_2023_29099_MOESM1_ESM.pdf]
