# Supplementary material for: Detection of coronaviruses in insectivorous bats of Fore-Caucasus, 2021
Source: Sci Rep. 2023 Feb 9;13:2306. doi: 10.1038/s41598-023-29099-6 (PMC9909659; doi:10.1038/s41598-023-29099-6)
Supplement: Supplementary file 4 — Supplementary Information 4. [file 41598_2023_29099_MOESM4_ESM.pdf]

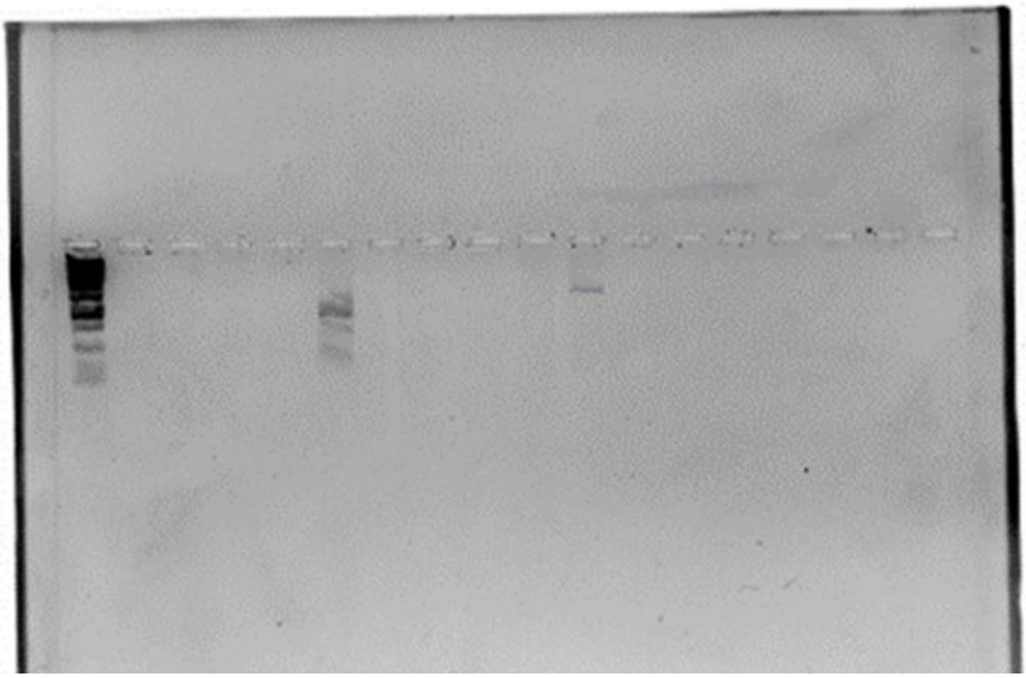

Figure S2. Results of gel electrophoresis of amplified cDNA of SARS-like CoVs' gRNA prepared with NEBNext ARTIC SARS-CoV-2 Library Prep Kit.
