# Supplementary material for: Detection of coronaviruses in insectivorous bats of Fore-Caucasus, 2021
Source: Sci Rep. 2023 Feb 9;13:2306. doi: 10.1038/s41598-023-29099-6 (PMC9909659; doi:10.1038/s41598-023-29099-6)
Supplement: Supplementary file 5 — Supplementary Information 5. [file 41598_2023_29099_MOESM5_ESM.pdf]

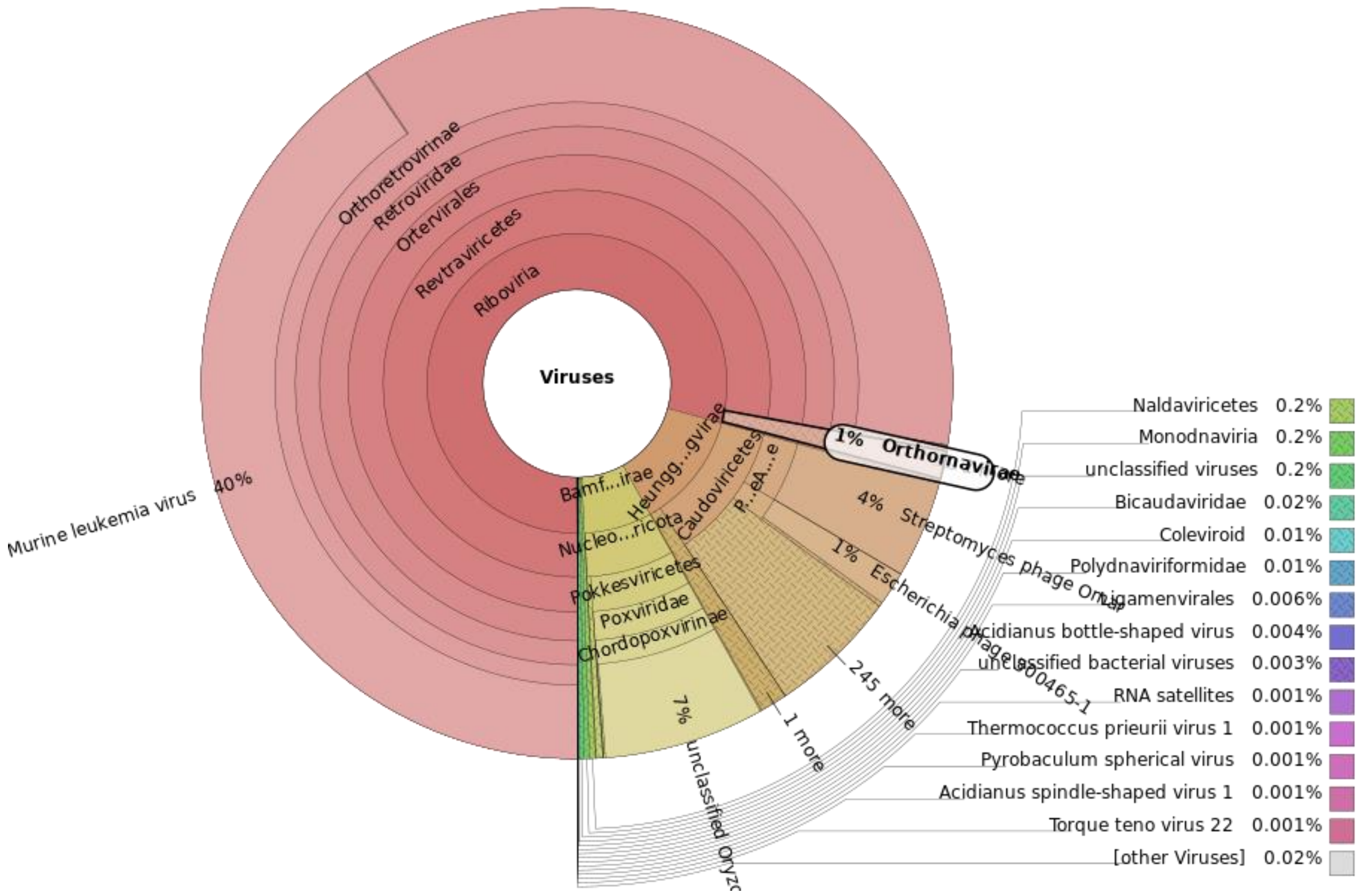


**Figure S3.** Taxonomical identification of viral reads from NovaSeq run.

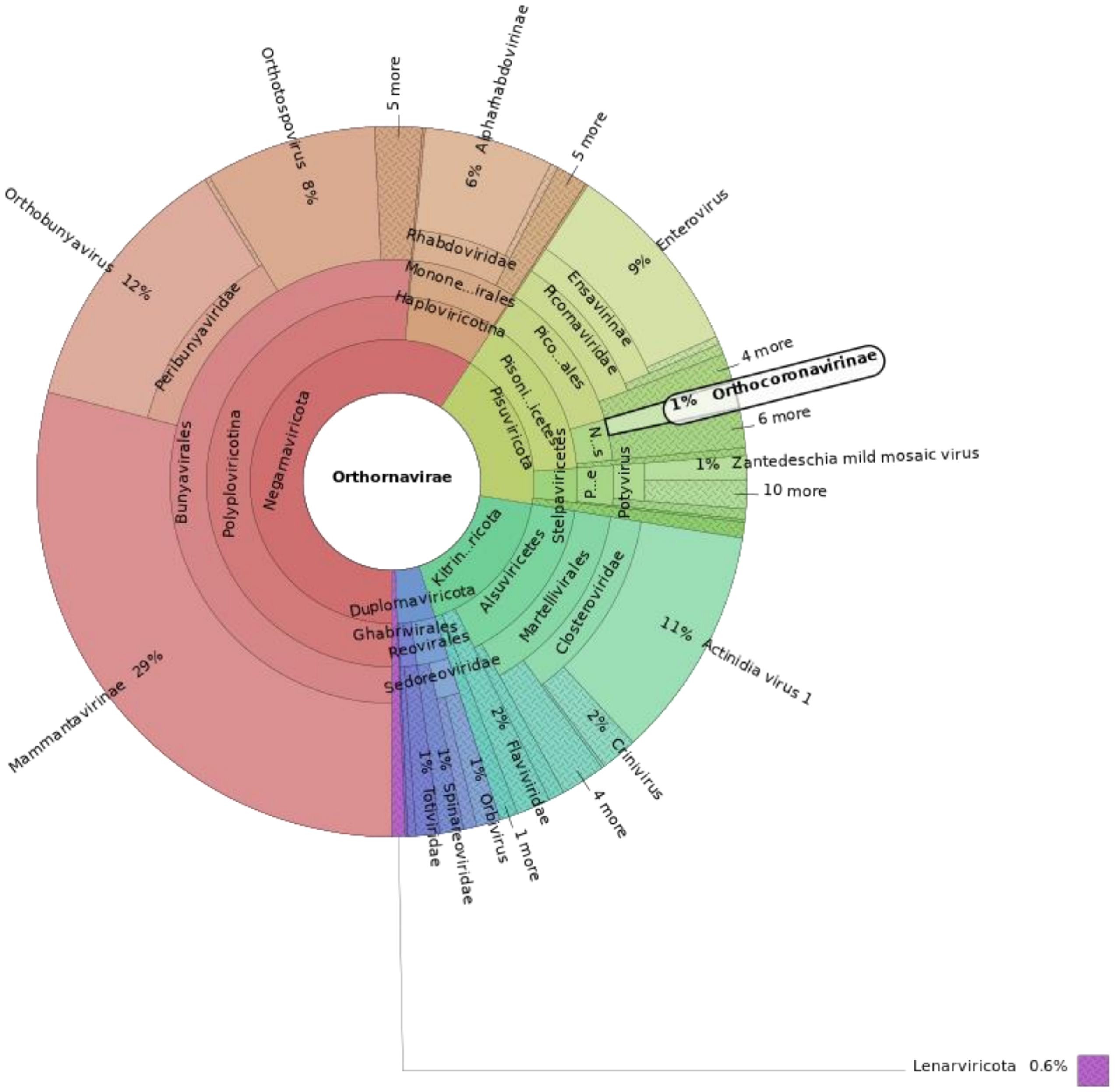


**Figure S4.** Taxonomical identification of ortornaviridae reads from NovaSeq run.

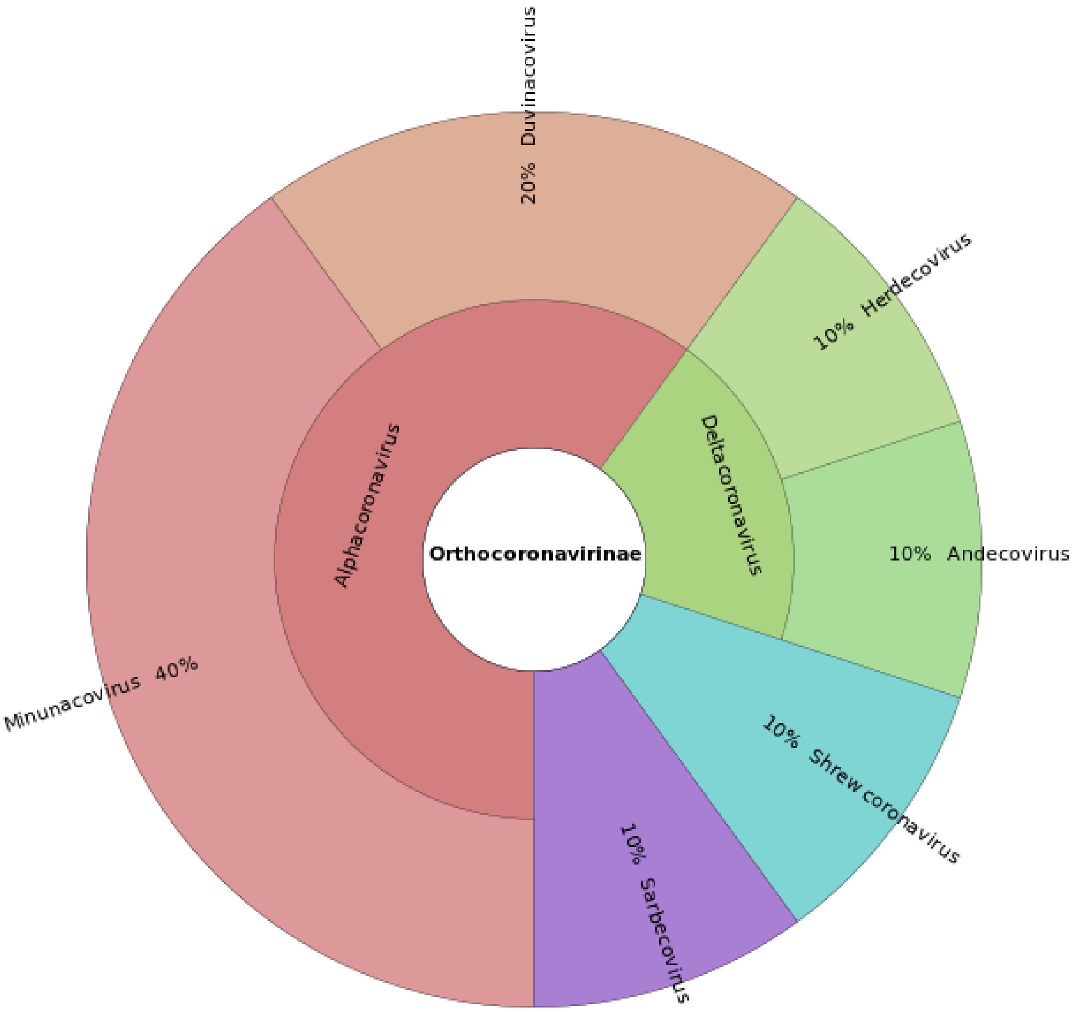


**Figure S5.** Taxonomical identification of *orthocoronavirinae* reads from NovaSeq run.

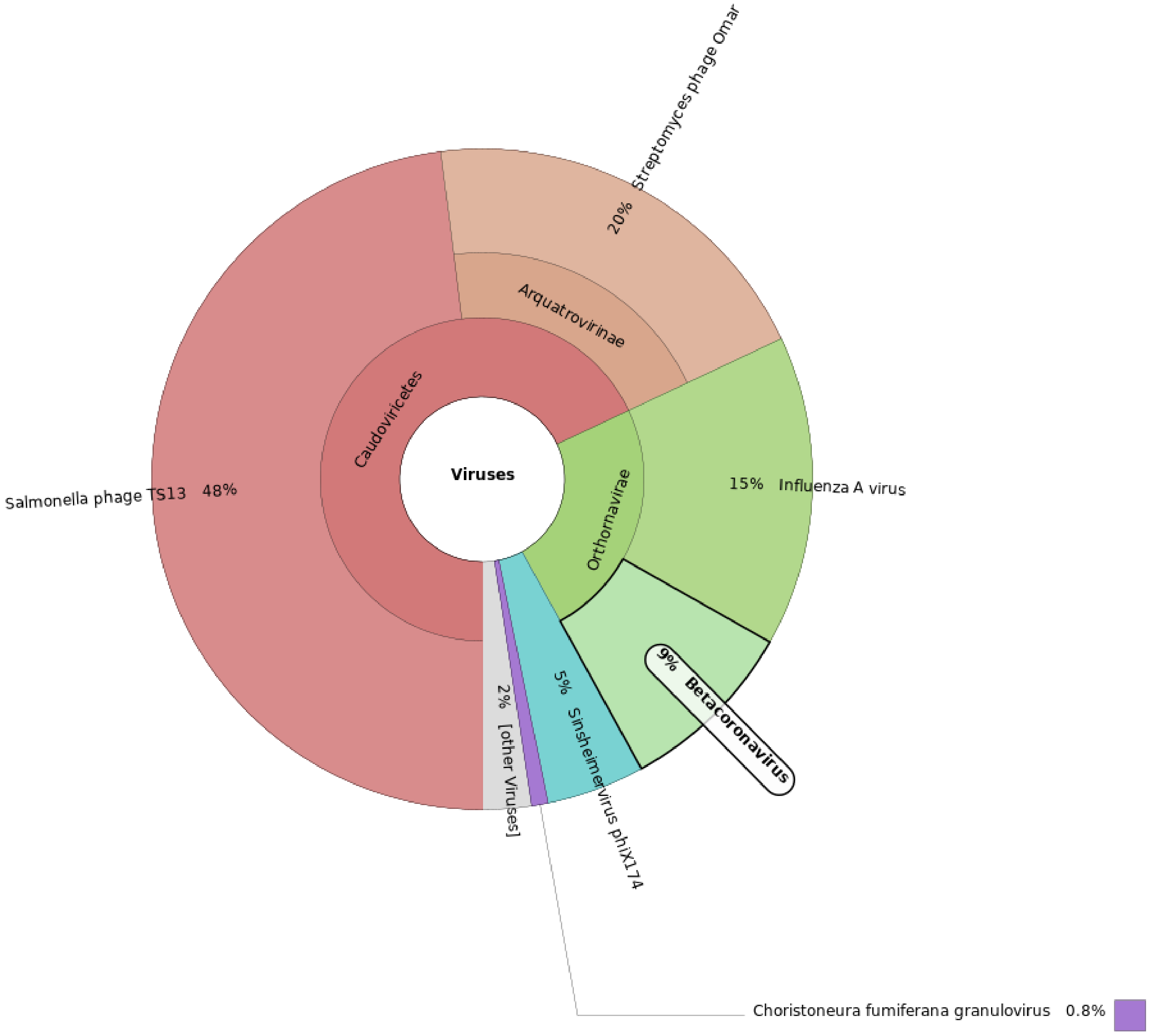


**Figure S6.** Taxonomical identification of viral reads from MiSeq run.
