# Supplementary material for: Detection of coronaviruses in insectivorous bats of Fore-Caucasus, 2021
Source: Sci Rep. 2023 Feb 9;13:2306. doi: 10.1038/s41598-023-29099-6 (PMC9909659; doi:10.1038/s41598-023-29099-6)
Supplement: Supplementary file 6 — Supplementary Information 6. [file 41598_2023_29099_MOESM6_ESM.pdf]

Supplementary File S5 contains assembled scaffolds from NGS raw data and results of BLAST search of similar viral nucleotide (ssRNA(+)) sequences.

Sample 1, *Pipistrellus kuhlii*, male, Adygea Republic, oropharyngeal swab.

>NODE_1_length_833_cov_17.774788
CCCTCCCCCCTCGCTACCACTCTACCCCTCACCGACGCTCTTCCGATCTGACCATGACGCGACCTCCACATAATGGGTCAGCGACTTATATTCTGTAGCGAGGTTAAC
CAATTAGGGGAGCCGTAGGGAAACCGAGTCTTAATAGGGCGAATTTAGTTGCAGGGTATAGACCCGAAACCGAGTGATCTATCCATGAGCAGGTTGAAAGTGCCG
TAACAGGCATCGGAGGACCGAACCCACTCCCGTTGAAAAGGTAGGGGATGACTTGTGGATAGGGGTGAAAGGCTAATCAAACTCGGTGATAGCTGGTTCTCCCCG
AAAGCTATTTAGGTAGCGCCTCGGACGAACACCATGAGGGGTGGAGGTCGCTAGATGGTCGAAGAACAAGTTTTGGAAAACTTGACTATAAAGGGTGATAGTCCC
GTATTTTAAATACAATGAGAAGCATATTAAGTAGTGCGGGACACGAGAAATCCTGTATGAAGATGGGGGGACCATCCTCCAAGGCTAAATACTCCTGACTGACCGA
TAGTGAACCAGTACCGTGAGGGAAAGGCGAAAAGAACCCCTGTGAGGGGAGTGAAATAGAACCTGAAACCGTATGCATACAAGCAGTGGGAGACCGTGGAGGTC
GCTAGATGGTCAGATCGGAAGAGCGTCGTGTAGGGAAAGAGTGTTACCGAGGGTGTAGATCTCGGTGTTGTTCTTGATATTAATATAATATCAACATCATCTACACT
GCTGTACGCTTTCCAATCTTCCAACATTACTATGTCTCATTCTCCTCTTTTTATTACCTTCTCTCTTCTTTTCATCTCCTATCTTATTCCTGC

Accession	Coverage	Identity	Release_Date	Species	Molecule_type	Length	Geo_Location	Host
MZ679046.1	11	89	2022-02-23T00:00:00Z	Sapovirus sp.	ssRNA(+)	7353	China: Xinjiang	
OV195202.1	6	91	2021-12-15T00:00:00Z	Severe acute respiratory syndrome-related coronavirus	ssRNA(+)	30221	Germany:Europe/Germany/Baden-Wurttemberg	Homo sapiens
OV195277.1	6	89	2021-12-15T00:00:00Z	Severe acute respiratory syndrome-related coronavirus	ssRNA(+)	30090	Germany:Europe/Germany/Baden-Wurttemberg	Homo sapiens
OV195597.1	4	100	2021-12-15T00:00:00Z	Severe acute respiratory syndrome-related coronavirus	ssRNA(+)	30117	Germany:Europe/Germany/Baden-Wurttemberg	Homo sapiens
OV195847.1	3	100	2021-12-15T00:00:00Z	Severe acute respiratory syndrome-related coronavirus	ssRNA(+)	30308	Germany:Europe/Germany/Baden-Wurttemberg	Homo sapiens
OV195900.1	6	89	2021-12-15T00:00:00Z	Severe acute respiratory syndrome-related coronavirus	ssRNA(+)	30109	Germany:Europe/Germany/Baden-Wurttemberg	Homo sapiens
OV195924.1	6	91	2021-12-15T00:00:00Z	Severe acute respiratory syndrome-related coronavirus	ssRNA(+)	30333	Germany:Europe/Germany/Baden-Wurttemberg	Homo sapiens
OV196000.1	4	100	2021-12-15T00:00:00Z	Severe acute respiratory syndrome-related coronavirus	ssRNA(+)	30106	Germany:Europe/Germany/Baden-Wurttemberg	Homo sapiens
OV196022.1	6	90	2021-12-15T00:00:00Z	Severe acute respiratory syndrome-related coronavirus	ssRNA(+)	30562	Germany:Europe/Germany/Baden-Wurttemberg	Homo sapiens
OV196096.1	6	91	2021-12-15T00:00:00Z	Severe acute respiratory syndrome-related coronavirus	ssRNA(+)	29646	Germany:Europe/Germany/Baden-Wurttemberg	Homo sapiens
OV196177.1	6	87	2021-12-15T00:00:00Z	Severe acute respiratory syndrome-related coronavirus	ssRNA(+)	30003	Germany:Europe/Germany/Baden-Wurttemberg	Homo sapiens
OV196273.1	6	89	2021-12-15T00:00:00Z	Severe acute respiratory syndrome-related coronavirus	ssRNA(+)	30444	Germany:Europe/Germany/Baden-Wurttemberg	Homo sapiens

OV196275.1	6	91	2021-12-15T00:00:00Z	Severe acute respiratory syndrome-related coronavirus	ssRNA(+)	30423	Germany:Europe/Germany/Baden-Wurttemberg	Homo sapiens
OV196298.1	6	91	2021-12-15T00:00:00Z	Severe acute respiratory syndrome-related coronavirus	ssRNA(+)	30559	Germany:Europe/Germany/Baden-Wurttemberg	Homo sapiens
OV196467.1	4	100	2021-12-15T00:00:00Z	Severe acute respiratory syndrome-related coronavirus	ssRNA(+)	30053	Germany:Europe/Germany/Baden-Wurttemberg	Homo sapiens
OV196488.1	6	89	2021-12-15T00:00:00Z	Severe acute respiratory syndrome-related coronavirus	ssRNA(+)	30094	Germany:Europe/Germany/Baden-Wurttemberg	Homo sapiens
OV196492.1	6	91	2021-12-15T00:00:00Z	Severe acute respiratory syndrome-related coronavirus	ssRNA(+)	30243	Germany:Europe/Germany/Baden-Wurttemberg	Homo sapiens
OV196499.1	6	89	2021-12-15T00:00:00Z	Severe acute respiratory syndrome-related coronavirus	ssRNA(+)	30525	Germany:Europe/Germany/Baden-Wurttemberg	Homo sapiens
OV196584.1	4	100	2021-12-15T00:00:00Z	Severe acute respiratory syndrome-related coronavirus	ssRNA(+)	30066	Germany:Europe/Germany/Baden-Wurttemberg	Homo sapiens
OV196890.1	6	89	2021-12-15T00:00:00Z	Severe acute respiratory syndrome-related coronavirus	ssRNA(+)	30101	Germany:Europe/Germany/Baden-Wurttemberg	Homo sapiens
OV197136.1	6	92	2021-12-15T00:00:00Z	Severe acute respiratory syndrome-related coronavirus	ssRNA(+)	30102	Germany:Europe/Germany/Baden-Wurttemberg	Homo sapiens
OV197187.1	6	93	2021-12-15T00:00:00Z	Severe acute respiratory syndrome-related coronavirus	ssRNA(+)	30208	Germany:Europe/Germany/Baden-Wurttemberg	Homo sapiens
OV197189.1	6	89	2021-12-15T00:00:00Z	Severe acute respiratory syndrome-related coronavirus	ssRNA(+)	30094	Germany:Europe/Germany/Baden-Wurttemberg	Homo sapiens
OV197539.1	6	91	2021-12-15T00:00:00Z	Severe acute respiratory syndrome-related coronavirus	ssRNA(+)	30057	Germany:Europe/Germany/Baden-Wurttemberg	Homo sapiens
OV197600.1	6	91	2021-12-15T00:00:00Z	Severe acute respiratory syndrome-related coronavirus	ssRNA(+)	30105	Germany:Europe/Germany/Baden-Wurttemberg	Homo sapiens
OV016109.1	6	91	2021-11-23T00:00:00Z	Severe acute respiratory syndrome-related coronavirus	ssRNA(+)	30090	Germany:Europe/Germany/Baden-Wurttemberg	Homo sapiens
LC315647.2	3	100	2017-08-09T00:00:00Z	Betacoronavirus 1	ssRNA(+)	30755	Japan: Tokyo	Homo sapiens

Sample 2, *Pipistrellus kuhlii*, Male, Krasnodar Krai, oropharyngeal swab.

>NODE_1_length_1236_cov_27.330027
TTTTGTTTGTTGTTTTTTTTTTTTTTTTTTTTTCAAGCAGAAGACGGCATACGAGATGTATTATGGTGACTGGAGTTCAGACGTGTGCTCCTTCCGATCTGACCATCTAGC
GACCTCCACGGCAAACCGGGAGAAGTGAAACATCTCAGTACCCCGAGGAAAAGACATCAATAGAGATTCCCTCAGTAGCGGCGAGCGAAAGGGGAAAAGCCGAC
GACTCATTGTATAGAAGAACAAGTTTTGGAAAACTTGACTATAAAGGGTGATAGTCCCGTATTTTAAATACAATGAGAAGCATATTAAGTAGTGCGGGACACGAGA
AATCCTGTATGAAGATGGGGGGACCATCCTCCAAGGCTAAATACTCCTGACTGACCGATAGTGAACCAGTACCGTGAGGGAAAGGCGAAAAGAACCCCTGTGAGG
GGAGTGAAATAGAACCTGAAACCGTATGCATACAAGCAGTGGGAGCCGTAAGGTGACCGCGTACCTTTTGTATAATGGGTCAGCGACTTATATTCTGTAGCGAGGT

TAACCAATTAGGGGAGCCGTAGGGAAACCGAGTCTTAATAGGGCGAATTTAGTTGCAGGGTATAGACCCGAAACCGAGTGATCTATCCATGAGCAGGTTGAAAGT
GCCGTAACAGGCATCGGAGGACCGAACCCACTCCCGTTGAAAAGGTAGGGGATGACTTGTGGATAGGGGTGAAAGGCTAATCAAACTCGGTGATAGCTGGTTCTC
CCCGAAAGCTATTTAGGTAGCGCCTCGGACGAACACCATGGGGGGTAGGTGGAGGTCGCTAGATGGTCGACCATCTAGCGACCTCCACTTGGCCCGGGAACGTATT
CACCGCGACATTCTGATCCGCGATTACTAGCGATTCCGACTTCATGGAGTCGAGTTGCAGACTCCAATCTGGACTACGATAGGCTTTTTGAGATGGAAGAGCACAC
GTCTGAACTCCAGTCACCATAATACATCTCGTATGCCGTCTTCTGCTTGAAAAAAAAAAAAAAAAAAAAAAAAAAAAAAAAAAAAAAAAAAAAAAAAAAAAA
AAATAACTTAGACACATATCCCTATAGTATTGTTATTAGCATGACGTGAGATAAAAGTCAAGTGACAAGACGGACATAAACAGATAATGACACTATGTGTTGTCAG
AGACAGTCATACCCACGAGCTGTCTCAAACAACATCACATGTAATATACTATGACAACACACTGATAACACAAA

Accession	Coverage	Identity	Release_Date	Species	Molecule_type	Length	Geo_Location	Host
NC_055577.1	2	96	2021-06-01T00:00:00Z	Physalis rugose mosaic virus	ssRNA(+)	4175	Brazil	Physalis peruviana
MZ679046.1	7	89	2022-02-23T00:00:00Z	Sapovirus sp.	ssRNA(+)	7353	China: Xinjiang	
OV377758.1	7	91	2022-01-09T00:00:00Z	Severe acute respiratory syndrome-related coronavirus	ssRNA(+)	30273	Germany:Europe/Germany/Baden-Wurttemberg	Homo sapiens
BK059322.1	2	97	2021-12-18T00:00:00Z	Arabis mosaic virus	ssRNA(+)	7408		Narcissus pseudonarcissus
OV195107.1	8	92	2021-12-15T00:00:00Z	Severe acute respiratory syndrome-related coronavirus	ssRNA(+)	30103	Germany:Europe/Germany/Baden-Wurttemberg	Homo sapiens
OV195202.1	6	89	2021-12-15T00:00:00Z	Severe acute respiratory syndrome-related coronavirus	ssRNA(+)	30221	Germany:Europe/Germany/Baden-Wurttemberg	Homo sapiens
OV195277.1	6	89	2021-12-15T00:00:00Z	Severe acute respiratory syndrome-related coronavirus	ssRNA(+)	30090	Germany:Europe/Germany/Baden-Wurttemberg	Homo sapiens
OV195597.1	6	91	2021-12-15T00:00:00Z	Severe acute respiratory syndrome-related coronavirus	ssRNA(+)	30117	Germany:Europe/Germany/Baden-Wurttemberg	Homo sapiens
OV195847.1	6	92	2021-12-15T00:00:00Z	Severe acute respiratory syndrome-related coronavirus	ssRNA(+)	30308	Germany:Europe/Germany/Baden-Wurttemberg	Homo sapiens
OV195900.1	5	88	2021-12-15T00:00:00Z	Severe acute respiratory syndrome-related coronavirus	ssRNA(+)	30109	Germany:Europe/Germany/Baden-Wurttemberg	Homo sapiens
OV195924.1	6	92	2021-12-15T00:00:00Z	Severe acute respiratory syndrome-related coronavirus	ssRNA(+)	30333	Germany:Europe/Germany/Baden-Wurttemberg	Homo sapiens
OV196000.1	6	92	2021-12-15T00:00:00Z	Severe acute respiratory syndrome-related coronavirus	ssRNA(+)	30106	Germany:Europe/Germany/Baden-Wurttemberg	Homo sapiens
OV196009.1	9	92	2021-12-15T00:00:00Z	Severe acute respiratory syndrome-related coronavirus	ssRNA(+)	30101	Germany:Europe/Germany/Baden-Wurttemberg	Homo sapiens
OV196022.1	15	95	2021-12-15T00:00:00Z	Severe acute respiratory syndrome-related coronavirus	ssRNA(+)	30562	Germany:Europe/Germany/Baden-Wurttemberg	Homo sapiens
OV196026.1	17	90	2021-12-15T00:00:00Z	Severe acute respiratory syndrome-related coronavirus	ssRNA(+)	30121	Germany:Europe/Germany/Baden-Wurttemberg	Homo sapiens

OV196099.1	6	92	2021-12-15T00:00:00Z	Severe acute respiratory syndrome-related coronavirus	ssRNA(+)	30100	Germany:Europe/Germany/Baden-Wurttemberg	Homo sapiens
OV196107.1	9	91	2021-12-15T00:00:00Z	Severe acute respiratory syndrome-related coronavirus	ssRNA(+)	30511	Germany:Europe/Germany/Baden-Wurttemberg	Homo sapiens
OV196177.1	6	92	2021-12-15T00:00:00Z	Severe acute respiratory syndrome-related coronavirus	ssRNA(+)	30003	Germany:Europe/Germany/Baden-Wurttemberg	Homo sapiens
OV196273.1	6	95	2021-12-15T00:00:00Z	Severe acute respiratory syndrome-related coronavirus	ssRNA(+)	30444	Germany:Europe/Germany/Baden-Wurttemberg	Homo sapiens
OV196275.1	6	92	2021-12-15T00:00:00Z	Severe acute respiratory syndrome-related coronavirus	ssRNA(+)	30423	Germany:Europe/Germany/Baden-Wurttemberg	Homo sapiens
OV196298.1	6	92	2021-12-15T00:00:00Z	Severe acute respiratory syndrome-related coronavirus	ssRNA(+)	30559	Germany:Europe/Germany/Baden-Wurttemberg	Homo sapiens
OV196467.1	5	93	2021-12-15T00:00:00Z	Severe acute respiratory syndrome-related coronavirus	ssRNA(+)	30053	Germany:Europe/Germany/Baden-Wurttemberg	Homo sapiens
OV196488.1	6	92	2021-12-15T00:00:00Z	Severe acute respiratory syndrome-related coronavirus	ssRNA(+)	30094	Germany:Europe/Germany/Baden-Wurttemberg	Homo sapiens
OV196492.1	6	92	2021-12-15T00:00:00Z	Severe acute respiratory syndrome-related coronavirus	ssRNA(+)	30243	Germany:Europe/Germany/Baden-Wurttemberg	Homo sapiens
OV196584.1	6	91	2021-12-15T00:00:00Z	Severe acute respiratory syndrome-related coronavirus	ssRNA(+)	30066	Germany:Europe/Germany/Baden-Wurttemberg	Homo sapiens
OV196626.1	6	92	2021-12-15T00:00:00Z	Severe acute respiratory syndrome-related coronavirus	ssRNA(+)	30095	Germany:Europe/Germany/Baden-Wurttemberg	Homo sapiens
OV196890.1	6	90	2021-12-15T00:00:00Z	Severe acute respiratory syndrome-related coronavirus	ssRNA(+)	30101	Germany:Europe/Germany/Baden-Wurttemberg	Homo sapiens
OV197136.1	6	90	2021-12-15T00:00:00Z	Severe acute respiratory syndrome-related coronavirus	ssRNA(+)	30102	Germany:Europe/Germany/Baden-Wurttemberg	Homo sapiens
OV197187.1	6	90	2021-12-15T00:00:00Z	Severe acute respiratory syndrome-related coronavirus	ssRNA(+)	30208	Germany:Europe/Germany/Baden-Wurttemberg	Homo sapiens
OV197189.1	6	95	2021-12-15T00:00:00Z	Severe acute respiratory syndrome-related coronavirus	ssRNA(+)	30094	Germany:Europe/Germany/Baden-Wurttemberg	Homo sapiens
OV197487.1	5	92	2021-12-15T00:00:00Z	Severe acute respiratory syndrome-related coronavirus	ssRNA(+)	30104	Germany:Europe/Germany/Baden-Wurttemberg	Homo sapiens
OV197539.1	6	93	2021-12-15T00:00:00Z	Severe acute respiratory syndrome-related coronavirus	ssRNA(+)	30057	Germany:Europe/Germany/Baden-Wurttemberg	Homo sapiens
OV197600.1	6	91	2021-12-15T00:00:00Z	Severe acute respiratory syndrome-related coronavirus	ssRNA(+)	30105	Germany:Europe/Germany/Baden-Wurttemberg	Homo sapiens
OV264044.1	10	92	2021-12-15T00:00:00Z	Severe acute respiratory syndrome-related coronavirus	ssRNA(+)	30466	Germany:Europe/Germany/Baden-Wurttemberg	Homo sapiens

OV015804.1	6	92	2021-11-23T00:00:00Z	Severe acute respiratory syndrome-related coronavirus	ssRNA(+)	29996	Germany:Europe/Germany/Baden-Wurttemberg	Homo sapiens
OV016108.1	6	94	2021-11-23T00:00:00Z	Severe acute respiratory syndrome-related coronavirus	ssRNA(+)	30390	Germany:Europe/Germany/Baden-Wurttemberg	Homo sapiens
OV016109.1	6	91	2021-11-23T00:00:00Z	Severe acute respiratory syndrome-related coronavirus	ssRNA(+)	30090	Germany:Europe/Germany/Baden-Wurttemberg	Homo sapiens
MK681145.1	2	96	2019-09-10T00:00:00Z	Physalis rugose mosaic virus	ssRNA(+)	4175	Brazil	Physalis peruviana

>NODE_8_length_458_cov_3.072508
GTTTGTCTATTGTGTAGTTTACTGCTGTGTCGTTGTTCAGATAATGTCTATGTCGTAGATTGAATTGGCAGTGTATCATTGCTCCTGTCTTTTTGTTATCTTTTTTTTTT
TCAAGCAGAAGACGGCATACGAGATGTATTATGGTGACTGGAGTTCAGACGTGTGCTCTTCAGTGAAGATGCTGTGTACCCGCGGCTAGACGGAAAGACCCCGTGA
ACCTTTACTACAGCTTTACATTGAACTTTGACCTTACCTGTGTAGGATAGGTGGGAGGCTTTGAAGTAGGGACGCCAGTTCCTATGGAGCCAACCTTGAAATACCAC
CCTGGTAATGTTGGGGTTCTAACTTAAGATAAACCAATCTAAGGACAATGTATGGTGGGTAGTTTGACTGGGGCGGTCTCCTCCTAAAGAGTAACGGAGGAGTACG
AAGGTGCACGTGGAGGTCGCTAGATGGTC

Accession	Coverage	Identity	Release_Date	Species	Molecule_type	Length	Geo_Location	Host
MZ679046.1	11	89	2022-02-23T00:00:00Z	Sapovirus sp.	ssRNA(+)	7353	China: Xinjiang	
OV195202.1	6	91	2021-12-15T00:00:00Z	Severe acute respiratory syndrome-related coronavirus	ssRNA(+)	30221	Germany:Europe/Germany/Baden-Wurttemberg	Homo sapiens
OV195277.1	6	89	2021-12-15T00:00:00Z	Severe acute respiratory syndrome-related coronavirus	ssRNA(+)	30090	Germany:Europe/Germany/Baden-Wurttemberg	Homo sapiens
OV195597.1	4	100	2021-12-15T00:00:00Z	Severe acute respiratory syndrome-related coronavirus	ssRNA(+)	30117	Germany:Europe/Germany/Baden-Wurttemberg	Homo sapiens
OV195847.1	3	100	2021-12-15T00:00:00Z	Severe acute respiratory syndrome-related coronavirus	ssRNA(+)	30308	Germany:Europe/Germany/Baden-Wurttemberg	Homo sapiens
OV195900.1	6	89	2021-12-15T00:00:00Z	Severe acute respiratory syndrome-related coronavirus	ssRNA(+)	30109	Germany:Europe/Germany/Baden-Wurttemberg	Homo sapiens
OV195924.1	6	91	2021-12-15T00:00:00Z	Severe acute respiratory syndrome-related coronavirus	ssRNA(+)	30333	Germany:Europe/Germany/Baden-Wurttemberg	Homo sapiens
OV196000.1	4	100	2021-12-15T00:00:00Z	Severe acute respiratory syndrome-related coronavirus	ssRNA(+)	30106	Germany:Europe/Germany/Baden-Wurttemberg	Homo sapiens
OV196022.1	6	90	2021-12-15T00:00:00Z	Severe acute respiratory syndrome-related coronavirus	ssRNA(+)	30562	Germany:Europe/Germany/Baden-Wurttemberg	Homo sapiens
OV196096.1	6	91	2021-12-15T00:00:00Z	Severe acute respiratory syndrome-related coronavirus	ssRNA(+)	29646	Germany:Europe/Germany/Baden-Wurttemberg	Homo sapiens
OV196177.1	6	87	2021-12-15T00:00:00Z	Severe acute respiratory syndrome-related coronavirus	ssRNA(+)	30003	Germany:Europe/Germany/Baden-Wurttemberg	Homo sapiens

OV196273.1	6	89	2021-12-15T00:00:00Z	Severe acute respiratory syndrome-related coronavirus	ssRNA(+)	30444	Germany:Europe/Germany/Baden-Wurttemberg	Homo sapiens
OV196275.1	6	91	2021-12-15T00:00:00Z	Severe acute respiratory syndrome-related coronavirus	ssRNA(+)	30423	Germany:Europe/Germany/Baden-Wurttemberg	Homo sapiens
OV196298.1	6	91	2021-12-15T00:00:00Z	Severe acute respiratory syndrome-related coronavirus	ssRNA(+)	30559	Germany:Europe/Germany/Baden-Wurttemberg	Homo sapiens
OV196467.1	4	100	2021-12-15T00:00:00Z	Severe acute respiratory syndrome-related coronavirus	ssRNA(+)	30053	Germany:Europe/Germany/Baden-Wurttemberg	Homo sapiens
OV196488.1	6	89	2021-12-15T00:00:00Z	Severe acute respiratory syndrome-related coronavirus	ssRNA(+)	30094	Germany:Europe/Germany/Baden-Wurttemberg	Homo sapiens
OV196492.1	6	91	2021-12-15T00:00:00Z	Severe acute respiratory syndrome-related coronavirus	ssRNA(+)	30243	Germany:Europe/Germany/Baden-Wurttemberg	Homo sapiens
OV196499.1	6	89	2021-12-15T00:00:00Z	Severe acute respiratory syndrome-related coronavirus	ssRNA(+)	30525	Germany:Europe/Germany/Baden-Wurttemberg	Homo sapiens
OV196584.1	4	100	2021-12-15T00:00:00Z	Severe acute respiratory syndrome-related coronavirus	ssRNA(+)	30066	Germany:Europe/Germany/Baden-Wurttemberg	Homo sapiens
OV196890.1	6	89	2021-12-15T00:00:00Z	Severe acute respiratory syndrome-related coronavirus	ssRNA(+)	30101	Germany:Europe/Germany/Baden-Wurttemberg	Homo sapiens
OV197136.1	6	92	2021-12-15T00:00:00Z	Severe acute respiratory syndrome-related coronavirus	ssRNA(+)	30102	Germany:Europe/Germany/Baden-Wurttemberg	Homo sapiens
OV197187.1	6	93	2021-12-15T00:00:00Z	Severe acute respiratory syndrome-related coronavirus	ssRNA(+)	30208	Germany:Europe/Germany/Baden-Wurttemberg	Homo sapiens
OV197189.1	6	89	2021-12-15T00:00:00Z	Severe acute respiratory syndrome-related coronavirus	ssRNA(+)	30094	Germany:Europe/Germany/Baden-Wurttemberg	Homo sapiens
OV197539.1	6	91	2021-12-15T00:00:00Z	Severe acute respiratory syndrome-related coronavirus	ssRNA(+)	30057	Germany:Europe/Germany/Baden-Wurttemberg	Homo sapiens
OV197600.1	6	91	2021-12-15T00:00:00Z	Severe acute respiratory syndrome-related coronavirus	ssRNA(+)	30105	Germany:Europe/Germany/Baden-Wurttemberg	Homo sapiens
OV016109.1	6	91	2021-11-23T00:00:00Z	Severe acute respiratory syndrome-related coronavirus	ssRNA(+)	30090	Germany:Europe/Germany/Baden-Wurttemberg	Homo sapiens
LC315647.2	3	100	2017-08-09T00:00:00Z	Betacoronavirus 1	ssRNA(+)	30755	Japan: Tokyo	Homo sapiens

Sample 3, *Eptesicus serotinus*, Male, Krasnodar Krai, oropharyngeal swab.

>NODE_2_length_747_cov_28.738710
GACCATCTAGCGACCTCCACCTGGCTCAGATTGAACGCTGGCGGCATGCTTTACACATGCAAGTCGAACGGCAGCGCGGGCTTCGGCCTGGCGGCGAGTGGCGAAC
GGGTGAGTAATACATCGGAACGTACCTGGTAGTGGGGGATAGCTCGGCGAAAGCCGGATTAATACCGCATACGACCTCAGGGTGAAAGCGGGGGACCTTCGGGCC
TCGCGCTATCAGAGCGGCCGATGGCAGATTAGCTGGTTGGTGAGGTAAAGGCTTACCAAGGCGACGATCTGTAGCTGGTCTGAGAGGACGACCAGCCACACTGGG

ACTGAGACACGGCCCAGACTCCTACGGGAGGCAGCAGTGGGGAATTTTGGACAATGGGCGCAAGCCTGATCCAGCAATGCCGCGTGTGTGATGAAGGCCTTCGGG
TTGTAAAGCACTTTTGTCAGGAACGAAAAGGTTCGTGTTAATACCATGGGCCGATGACGGTACCTGAAGAATAAGCACCGGCTAACTACGTGCCAGCAGCCGCGGT
AATACGTAGGGTGCGAGCGTTAATCGGAATTACTGGGCGTAAAGCGTGCGCAGGCGGTTTTGCAAGACCGATGTGAAATCCCCGGGCTTAACCTGGGAACTGCATT
GGTGACTGCAAGGCTAGAGTGTGTCAGAGTGGAGGTCGGAAGAGCACACGTCTGAACTCCAGTCACGATCTATCATCTCGTATGCCGGCTTCTGCTTGAAAACAAA
AACAAACC

Accession	Coverage	Identity	Release_Date	Species	Molecule_type	Length	Host
NC_055577.1	4	100	2021-06-01T00:00:00Z	Physalis rugose mosaic virus	ssRNA(+)	4175	Physalis peruviana
OV377758.1	10	90	2022-01-09T00:00:00Z	Severe acute respiratory syndrome-related coronavirus	ssRNA(+)	30273	Homo sapiens
BK059322.1	4	100	2021-12-18T00:00:00Z	Arabis mosaic virus	ssRNA(+)	7408	Narcissus pseudonarcissus
OV195107.1	11	89	2021-12-15T00:00:00Z	Severe acute respiratory syndrome-related coronavirus	ssRNA(+)	30103	Homo sapiens
OV195202.1	9	89	2021-12-15T00:00:00Z	Severe acute respiratory syndrome-related coronavirus	ssRNA(+)	30221	Homo sapiens
OV195277.1	9	90	2021-12-15T00:00:00Z	Severe acute respiratory syndrome-related coronavirus	ssRNA(+)	30090	Homo sapiens
OV195597.1	10	88	2021-12-15T00:00:00Z	Severe acute respiratory syndrome-related coronavirus	ssRNA(+)	30117	Homo sapiens
OV195847.1	10	88	2021-12-15T00:00:00Z	Severe acute respiratory syndrome-related coronavirus	ssRNA(+)	30308	Homo sapiens
OV195900.1	8	86	2021-12-15T00:00:00Z	Severe acute respiratory syndrome-related coronavirus	ssRNA(+)	30109	Homo sapiens
OV195924.1	10	91	2021-12-15T00:00:00Z	Severe acute respiratory syndrome-related coronavirus	ssRNA(+)	30333	Homo sapiens
OV196000.1	10	92	2021-12-15T00:00:00Z	Severe acute respiratory syndrome-related coronavirus	ssRNA(+)	30106	Homo sapiens
OV196009.1	11	88	2021-12-15T00:00:00Z	Severe acute respiratory syndrome-related coronavirus	ssRNA(+)	30101	Homo sapiens
OV196022.1	10	91	2021-12-15T00:00:00Z	Severe acute respiratory syndrome-related coronavirus	ssRNA(+)	30562	Homo sapiens
OV196026.1	11	88	2021-12-15T00:00:00Z	Severe acute respiratory syndrome-related coronavirus	ssRNA(+)	30121	Homo sapiens
OV196099.1	10	88	2021-12-15T00:00:00Z	Severe acute respiratory syndrome-related coronavirus	ssRNA(+)	30100	Homo sapiens
OV196107.1	11	89	2021-12-15T00:00:00Z	Severe acute respiratory syndrome-related coronavirus	ssRNA(+)	30511	Homo sapiens

OV196177.1	10	89	2021-12-15T00:00:00Z	Severe acute respiratory syndrome-related coronavirus	ssRNA(+)	30003	Homo sapiens
OV196273.1	10	89	2021-12-15T00:00:00Z	Severe acute respiratory syndrome-related coronavirus	ssRNA(+)	30444	Homo sapiens
OV196275.1	10	89	2021-12-15T00:00:00Z	Severe acute respiratory syndrome-related coronavirus	ssRNA(+)	30423	Homo sapiens
OV196298.1	10	89	2021-12-15T00:00:00Z	Severe acute respiratory syndrome-related coronavirus	ssRNA(+)	30559	Homo sapiens
OV196467.1	8	91	2021-12-15T00:00:00Z	Severe acute respiratory syndrome-related coronavirus	ssRNA(+)	30053	Homo sapiens
OV196488.1	10	88	2021-12-15T00:00:00Z	Severe acute respiratory syndrome-related coronavirus	ssRNA(+)	30094	Homo sapiens
OV196492.1	10	89	2021-12-15T00:00:00Z	Severe acute respiratory syndrome-related coronavirus	ssRNA(+)	30243	Homo sapiens
OV196584.1	9	91	2021-12-15T00:00:00Z	Severe acute respiratory syndrome-related coronavirus	ssRNA(+)	30066	Homo sapiens
OV196626.1	10	93	2021-12-15T00:00:00Z	Severe acute respiratory syndrome-related coronavirus	ssRNA(+)	30095	Homo sapiens
OV196890.1	10	88	2021-12-15T00:00:00Z	Severe acute respiratory syndrome-related coronavirus	ssRNA(+)	30101	Homo sapiens
OV197136.1	10	91	2021-12-15T00:00:00Z	Severe acute respiratory syndrome-related coronavirus	ssRNA(+)	30102	Homo sapiens
OV197187.1	10	92	2021-12-15T00:00:00Z	Severe acute respiratory syndrome-related coronavirus	ssRNA(+)	30208	Homo sapiens
OV197189.1	10	91	2021-12-15T00:00:00Z	Severe acute respiratory syndrome-related coronavirus	ssRNA(+)	30094	Homo sapiens
OV197487.1	8	92	2021-12-15T00:00:00Z	Severe acute respiratory syndrome-related coronavirus	ssRNA(+)	30104	Homo sapiens
OV197539.1	9	90	2021-12-15T00:00:00Z	Severe acute respiratory syndrome-related coronavirus	ssRNA(+)	30057	Homo sapiens
OV197600.1	10	91	2021-12-15T00:00:00Z	Severe acute respiratory syndrome-related coronavirus	ssRNA(+)	30105	Homo sapiens
OV264044.1	10	88	2021-12-15T00:00:00Z	Severe acute respiratory syndrome-related coronavirus	ssRNA(+)	30466	Homo sapiens
OV015804.1	10	88	2021-11-23T00:00:00Z	Severe acute respiratory syndrome-related coronavirus	ssRNA(+)	29996	Homo sapiens
OV016108.1	10	89	2021-11-23T00:00:00Z	Severe acute respiratory syndrome-related coronavirus	ssRNA(+)	30390	Homo sapiens

OV016109.1	10	89	2021-11-23T00:00:00Z	Severe acute respiratory syndrome-related coronavirus	ssRNA(+)	30090	Homo sapiens
MK681145.1	4	100	2019-09-10T00:00:00Z	Physalis rugose mosaic virus	ssRNA(+)	4175	Physalis peruviana

>NODE_6_length_499_cov_1.381720
CGTGCTCGTTAGTTTTGAAGCTACTACGCCGCTAGCTAAGCGTACAGCATGAGGCTACACTCTTTCCCTACACGACGCTCTTCCGATCTGACCATCTAGCGACCTCC
ACGGTACTCATCCGCTATCGGTCACACTAAGTATTTAGGCTTACCGGGTGGTCCCGGCAGATTCACAGCAGATTCCACGAGCCCGCTGCTACTCGGGCACCCAACCA
ACCACACGCACACGCATATTCACGTACAGGACTCTCACCTTCTACAGTGGGCGATTCCACACCACTTCCGCTTACACACACACGCACAGCAAAACGTCAAGAGCAC
ACGTCTGAACTCCAGTCACGATCTATCATCTCGTATGCCGTCTTCTGCTTGAAAAAAAAAAAAAAAATAACAACAATCACGCAAGTAGAGTAAGCGATGCATCCAG
AGCGAAGGTAAAGAGAACAAGTTACAGAGCGAATACACACGACAATGAAAATACGAAAAGCACACGAAGAGCA

Accession	Coverage	Identity	Release_Date	Species	Molecule_type	Length	Geo_Location	Host
OV377758.1	15	94	2022-01-09T00:00:00Z	Severe acute respiratory syndrome-related coronavirus	ssRNA(+)	30273	Germany:Europe/Germany/Baden-Wurttemberg	Homo sapiens
BK059322.1	5	100	2021-12-18T00:00:00Z	Arabis mosaic virus	ssRNA(+)	7408		Narcissus pseudonarcissus
OV195107.1	15	93	2021-12-15T00:00:00Z	Severe acute respiratory syndrome-related coronavirus	ssRNA(+)	30103	Germany:Europe/Germany/Baden-Wurttemberg	Homo sapiens
OV195202.1	7	100	2021-12-15T00:00:00Z	Severe acute respiratory syndrome-related coronavirus	ssRNA(+)	30221	Germany:Europe/Germany/Baden-Wurttemberg	Homo sapiens
OV195277.1	21	92	2021-12-15T00:00:00Z	Severe acute respiratory syndrome-related coronavirus	ssRNA(+)	30090	Germany:Europe/Germany/Baden-Wurttemberg	Homo sapiens
OV195597.1	21	92	2021-12-15T00:00:00Z	Severe acute respiratory syndrome-related coronavirus	ssRNA(+)	30117	Germany:Europe/Germany/Baden-Wurttemberg	Homo sapiens
OV195847.1	6	100	2021-12-15T00:00:00Z	Severe acute respiratory syndrome-related coronavirus	ssRNA(+)	30308	Germany:Europe/Germany/Baden-Wurttemberg	Homo sapiens
OV195900.1	6	100	2021-12-15T00:00:00Z	Severe acute respiratory syndrome-related coronavirus	ssRNA(+)	30109	Germany:Europe/Germany/Baden-Wurttemberg	Homo sapiens
OV195924.1	21	94	2021-12-15T00:00:00Z	Severe acute respiratory syndrome-related coronavirus	ssRNA(+)	30333	Germany:Europe/Germany/Baden-Wurttemberg	Homo sapiens
OV196000.1	21	95	2021-12-15T00:00:00Z	Severe acute respiratory syndrome-related coronavirus	ssRNA(+)	30106	Germany:Europe/Germany/Baden-Wurttemberg	Homo sapiens
OV196022.1	6	100	2021-12-15T00:00:00Z	Severe acute respiratory syndrome-related coronavirus	ssRNA(+)	30562	Germany:Europe/Germany/Baden-Wurttemberg	Homo sapiens
OV196096.1	6	100	2021-12-15T00:00:00Z	Severe acute respiratory syndrome-related coronavirus	ssRNA(+)	29646	Germany:Europe/Germany/Baden-Wurttemberg	Homo sapiens
OV196107.1	14	93	2021-12-15T00:00:00Z	Severe acute respiratory syndrome-related coronavirus	ssRNA(+)	30511	Germany:Europe/Germany/Baden-Wurttemberg	Homo sapiens

OV196177.1	7	100	2021-12-15T00:00:00Z	Severe acute respiratory syndrome-related coronavirus	ssRNA(+)	30003	Germany:Europe/Germany/Baden-Wurttemberg	Homo sapiens
OV196273.1	6	100	2021-12-15T00:00:00Z	Severe acute respiratory syndrome-related coronavirus	ssRNA(+)	30444	Germany:Europe/Germany/Baden-Wurttemberg	Homo sapiens
OV196275.1	6	100	2021-12-15T00:00:00Z	Severe acute respiratory syndrome-related coronavirus	ssRNA(+)	30423	Germany:Europe/Germany/Baden-Wurttemberg	Homo sapiens
OV196298.1	7	100	2021-12-15T00:00:00Z	Severe acute respiratory syndrome-related coronavirus	ssRNA(+)	30559	Germany:Europe/Germany/Baden-Wurttemberg	Homo sapiens
OV196467.1	6	100	2021-12-15T00:00:00Z	Severe acute respiratory syndrome-related coronavirus	ssRNA(+)	30053	Germany:Europe/Germany/Baden-Wurttemberg	Homo sapiens
OV196488.1	6	100	2021-12-15T00:00:00Z	Severe acute respiratory syndrome-related coronavirus	ssRNA(+)	30094	Germany:Europe/Germany/Baden-Wurttemberg	Homo sapiens
OV196492.1	7	97	2021-12-15T00:00:00Z	Severe acute respiratory syndrome-related coronavirus	ssRNA(+)	30243	Germany:Europe/Germany/Baden-Wurttemberg	Homo sapiens
OV196499.1	6	100	2021-12-15T00:00:00Z	Severe acute respiratory syndrome-related coronavirus	ssRNA(+)	30525	Germany:Europe/Germany/Baden-Wurttemberg	Homo sapiens
OV196584.1	20	94	2021-12-15T00:00:00Z	Severe acute respiratory syndrome-related coronavirus	ssRNA(+)	30066	Germany:Europe/Germany/Baden-Wurttemberg	Homo sapiens
OV196626.1	13	95	2021-12-15T00:00:00Z	Severe acute respiratory syndrome-related coronavirus	ssRNA(+)	30095	Germany:Europe/Germany/Baden-Wurttemberg	Homo sapiens
OV196890.1	6	100	2021-12-15T00:00:00Z	Severe acute respiratory syndrome-related coronavirus	ssRNA(+)	30101	Germany:Europe/Germany/Baden-Wurttemberg	Homo sapiens
OV197136.1	22	93	2021-12-15T00:00:00Z	Severe acute respiratory syndrome-related coronavirus	ssRNA(+)	30102	Germany:Europe/Germany/Baden-Wurttemberg	Homo sapiens
OV197187.1	7	100	2021-12-15T00:00:00Z	Severe acute respiratory syndrome-related coronavirus	ssRNA(+)	30208	Germany:Europe/Germany/Baden-Wurttemberg	Homo sapiens
OV197189.1	22	93	2021-12-15T00:00:00Z	Severe acute respiratory syndrome-related coronavirus	ssRNA(+)	30094	Germany:Europe/Germany/Baden-Wurttemberg	Homo sapiens
OV197487.1	17	93	2021-12-15T00:00:00Z	Severe acute respiratory syndrome-related coronavirus	ssRNA(+)	30104	Germany:Europe/Germany/Baden-Wurttemberg	Homo sapiens
OV197539.1	6	100	2021-12-15T00:00:00Z	Severe acute respiratory syndrome-related coronavirus	ssRNA(+)	30057	Germany:Europe/Germany/Baden-Wurttemberg	Homo sapiens
OV197600.1	21	94	2021-12-15T00:00:00Z	Severe acute respiratory syndrome-related coronavirus	ssRNA(+)	30105	Germany:Europe/Germany/Baden-Wurttemberg	Homo sapiens
OV016109.1	22	93	2021-11-23T00:00:00Z	Severe acute respiratory syndrome-related coronavirus	ssRNA(+)	30090	Germany:Europe/Germany/Baden-Wurttemberg	Homo sapiens
LC315647.2	7	100	2017-08-09T00:00:00Z	Betacoronavirus 1	ssRNA(+)	30755	Japan: Tokyo	Homo sapiens

>NODE_9_length_466_cov_0.852507
GACCATCTAGCGACCTCCACCCAAATCCACAGCTCTTGATGTCTGTCACTTCCGTCTCTGGAAGGTAGCTGTGGATTTGGGGGTAAACACAACGCAGTTGGGTGATG
AAGGGCGGGTGACCCTGAGGGTACCCCACTTGCCAGCCCCGACTGTGGGGTGAGGTCCTCAAGTGTCAGGCAATGGAAAAGTGGAGGTCGCTAGATGGTCAAGAG
CACACGTCTGAACTCCAGTCACGATCTATCATCTCGTATGCCGTCTTCTGCTTGAAAAAAAAAAAAACAACAGAAACAAAAAAGCCACCTTCTAAGACTGTTCCAA
CCTACACCCGCTATGTTAACTCCATATGTTCAATGTAAACAACAGCTACCCCTTCATAATCAATGATAAACACGACATTACAACATAAAACATAACAGAATGCACC
ATTTATACAGAAAATACGCATCATAGCACATTAGTAAACACC

Accession	Coverage	Identity	Release_Date	Species	Molecule_type	Length	Geo_Location	Host
OV377758.1	16	93	2022-01-09T00:00:00Z	Severe acute respiratory syndrome-related coronavirus	ssRNA(+)	30273	Germany:Europe/Germany/Baden-Wurttemberg	Homo sapiens
BK059322.1	6	100	2021-12-18T00:00:00Z	Arabis mosaic virus	ssRNA(+)	7408		Narcissus pseudonarcissus
OV195107.1	18	91	2021-12-15T00:00:00Z	Severe acute respiratory syndrome-related coronavirus	ssRNA(+)	30103	Germany:Europe/Germany/Baden-Wurttemberg	Homo sapiens
OV195277.1	15	92	2021-12-15T00:00:00Z	Severe acute respiratory syndrome-related coronavirus	ssRNA(+)	30090	Germany:Europe/Germany/Baden-Wurttemberg	Homo sapiens
OV195597.1	16	90	2021-12-15T00:00:00Z	Severe acute respiratory syndrome-related coronavirus	ssRNA(+)	30117	Germany:Europe/Germany/Baden-Wurttemberg	Homo sapiens
OV195924.1	15	94	2021-12-15T00:00:00Z	Severe acute respiratory syndrome-related coronavirus	ssRNA(+)	30333	Germany:Europe/Germany/Baden-Wurttemberg	Homo sapiens
OV196000.1	15	95	2021-12-15T00:00:00Z	Severe acute respiratory syndrome-related coronavirus	ssRNA(+)	30106	Germany:Europe/Germany/Baden-Wurttemberg	Homo sapiens
OV196107.1	15	93	2021-12-15T00:00:00Z	Severe acute respiratory syndrome-related coronavirus	ssRNA(+)	30511	Germany:Europe/Germany/Baden-Wurttemberg	Homo sapiens
OV196584.1	15	94	2021-12-15T00:00:00Z	Severe acute respiratory syndrome-related coronavirus	ssRNA(+)	30066	Germany:Europe/Germany/Baden-Wurttemberg	Homo sapiens
OV196626.1	14	95	2021-12-15T00:00:00Z	Severe acute respiratory syndrome-related coronavirus	ssRNA(+)	30095	Germany:Europe/Germany/Baden-Wurttemberg	Homo sapiens
OV197136.1	16	93	2021-12-15T00:00:00Z	Severe acute respiratory syndrome-related coronavirus	ssRNA(+)	30102	Germany:Europe/Germany/Baden-Wurttemberg	Homo sapiens
OV197189.1	16	93	2021-12-15T00:00:00Z	Severe acute respiratory syndrome-related coronavirus	ssRNA(+)	30094	Germany:Europe/Germany/Baden-Wurttemberg	Homo sapiens
OV197487.1	12	93	2021-12-15T00:00:00Z	Severe acute respiratory syndrome-related coronavirus	ssRNA(+)	30104	Germany:Europe/Germany/Baden-Wurttemberg	Homo sapiens
OV197600.1	15	94	2021-12-15T00:00:00Z	Severe acute respiratory syndrome-related coronavirus	ssRNA(+)	30105	Germany:Europe/Germany/Baden-Wurttemberg	Homo sapiens
OV016109.1	15	93	2021-11-23T00:00:00Z	Severe acute respiratory syndrome-related coronavirus	ssRNA(+)	30090	Germany:Europe/Germany/Baden-Wurttemberg	Homo sapiens

Sample 4, *Eptesicus serotinus*, Female, Krasnodar Krai, oropharyngeal swab.

>NODE_1_length_707_cov_4.824138
CCTCTGACTCGCAGCGTAGTCTGATGTGAGGTTAGTACATGTTGTGATCGTGTCGTATGTGCGGTGGTTACATGATGGTGATGTATAGAGCAGCTGGGAGTAGTACG
TGACTTGGACATTATCGACGATCTATCTCACTGTGTGTGTTATAATAATGCTTTGGCTGCGTAATTGCATTTTTGAGTGTGTGTATATCACATCAGTGGCTTTTAGTG
CTATATTCTTTTTTTTTTTTTAATGACACGGCGACCACCGAGATCTACACGCAGAATCACACTCTTTCCCTACACGACGCTCTTCCGATCTGACCATCTAGCGACCT
CCACGAATGGTGTAACGATCTGGACACTGTCTCAGCCATGAGCTCGGTGAAATTGTAGTATCGGTGAAGATGCCGATTACCCGCAGTGGGACGAAAAGACCCTGTG
CACCTTTACTATAGCTTCGTATTGACCTTGGATAAGTGATGTGTAGGATAGGTGGGAGACTAAGAATCGGGTTCGCCAGGATTCGAGGAGTCATTGTTGAAATACC
ACCCTTTGCTTATCTGAGGCCTAACTCTCTTATAAGAGAGGACATTGCGTGGTGGGTAGTTTGACTGGGGTGGTCGCCTCCAAAAGCGTAACGGAGGCTTCTAAAGG
TTCCCTCAGCACGCTTGGTAACCGTGCGTAGAGTGCAATGGCAAAGTGGAGTTAGCTGGATGGTC

Accession	Coverage	Identity	Release_Date	Species	Molecule_type	Length	Geo_Location	Host
OV195202.1	12	92	2021-12-15T00:00:00Z	Severe acute respiratory syndrome-related coronavirus	ssRNA(+)	30221	Germany:Europe/Germany/Baden-Wurttemberg	Homo sapiens
OV195277.1	12	91	2021-12-15T00:00:00Z	Severe acute respiratory syndrome-related coronavirus	ssRNA(+)	30090	Germany:Europe/Germany/Baden-Wurttemberg	Homo sapiens
OV195597.1	8	93	2021-12-15T00:00:00Z	Severe acute respiratory syndrome-related coronavirus	ssRNA(+)	30117	Germany:Europe/Germany/Baden-Wurttemberg	Homo sapiens
OV195847.1	4	100	2021-12-15T00:00:00Z	Severe acute respiratory syndrome-related coronavirus	ssRNA(+)	30308	Germany:Europe/Germany/Baden-Wurttemberg	Homo sapiens
OV195900.1	12	95	2021-12-15T00:00:00Z	Severe acute respiratory syndrome-related coronavirus	ssRNA(+)	30109	Germany:Europe/Germany/Baden-Wurttemberg	Homo sapiens
OV195924.1	12	91	2021-12-15T00:00:00Z	Severe acute respiratory syndrome-related coronavirus	ssRNA(+)	30333	Germany:Europe/Germany/Baden-Wurttemberg	Homo sapiens
OV196000.1	4	100	2021-12-15T00:00:00Z	Severe acute respiratory syndrome-related coronavirus	ssRNA(+)	30106	Germany:Europe/Germany/Baden-Wurttemberg	Homo sapiens
OV196022.1	7	90	2021-12-15T00:00:00Z	Severe acute respiratory syndrome-related coronavirus	ssRNA(+)	30562	Germany:Europe/Germany/Baden-Wurttemberg	Homo sapiens
OV196096.1	8	88	2021-12-15T00:00:00Z	Severe acute respiratory syndrome-related coronavirus	ssRNA(+)	29646	Germany:Europe/Germany/Baden-Wurttemberg	Homo sapiens
OV196177.1	12	87	2021-12-15T00:00:00Z	Severe acute respiratory syndrome-related coronavirus	ssRNA(+)	30003	Germany:Europe/Germany/Baden-Wurttemberg	Homo sapiens
OV196273.1	12	89	2021-12-15T00:00:00Z	Severe acute respiratory syndrome-related coronavirus	ssRNA(+)	30444	Germany:Europe/Germany/Baden-Wurttemberg	Homo sapiens
OV196275.1	12	94	2021-12-15T00:00:00Z	Severe acute respiratory syndrome-related coronavirus	ssRNA(+)	30423	Germany:Europe/Germany/Baden-Wurttemberg	Homo sapiens
OV196298.1	12	93	2021-12-15T00:00:00Z	Severe acute respiratory syndrome-related coronavirus	ssRNA(+)	30559	Germany:Europe/Germany/Baden-Wurttemberg	Homo sapiens

OV196467.1	6	91	2021-12-15T00:00:00Z	Severe acute respiratory syndrome-related coronavirus	ssRNA(+)	30053	Germany:Europe/Germany/Baden-Wurttemberg	Homo sapiens
OV196488.1	12	92	2021-12-15T00:00:00Z	Severe acute respiratory syndrome-related coronavirus	ssRNA(+)	30094	Germany:Europe/Germany/Baden-Wurttemberg	Homo sapiens
OV196492.1	7	89	2021-12-15T00:00:00Z	Severe acute respiratory syndrome-related coronavirus	ssRNA(+)	30243	Germany:Europe/Germany/Baden-Wurttemberg	Homo sapiens
OV196499.1	12	91	2021-12-15T00:00:00Z	Severe acute respiratory syndrome-related coronavirus	ssRNA(+)	30525	Germany:Europe/Germany/Baden-Wurttemberg	Homo sapiens
OV196584.1	4	100	2021-12-15T00:00:00Z	Severe acute respiratory syndrome-related coronavirus	ssRNA(+)	30066	Germany:Europe/Germany/Baden-Wurttemberg	Homo sapiens
OV196890.1	8	93	2021-12-15T00:00:00Z	Severe acute respiratory syndrome-related coronavirus	ssRNA(+)	30101	Germany:Europe/Germany/Baden-Wurttemberg	Homo sapiens
OV197136.1	12	93	2021-12-15T00:00:00Z	Severe acute respiratory syndrome-related coronavirus	ssRNA(+)	30102	Germany:Europe/Germany/Baden-Wurttemberg	Homo sapiens
OV197187.1	12	87	2021-12-15T00:00:00Z	Severe acute respiratory syndrome-related coronavirus	ssRNA(+)	30208	Germany:Europe/Germany/Baden-Wurttemberg	Homo sapiens
OV197189.1	12	93	2021-12-15T00:00:00Z	Severe acute respiratory syndrome-related coronavirus	ssRNA(+)	30094	Germany:Europe/Germany/Baden-Wurttemberg	Homo sapiens
OV197539.1	12	95	2021-12-15T00:00:00Z	Severe acute respiratory syndrome-related coronavirus	ssRNA(+)	30057	Germany:Europe/Germany/Baden-Wurttemberg	Homo sapiens
OV197600.1	12	93	2021-12-15T00:00:00Z	Severe acute respiratory syndrome-related coronavirus	ssRNA(+)	30105	Germany:Europe/Germany/Baden-Wurttemberg	Homo sapiens
OV016109.1	12	93	2021-11-23T00:00:00Z	Severe acute respiratory syndrome-related coronavirus	ssRNA(+)	30090	Germany:Europe/Germany/Baden-Wurttemberg	Homo sapiens
LC315647.2	5	97	2017-08-09T00:00:00Z	Betacoronavirus 1	ssRNA(+)	30755	Japan: Tokyo	Homo sapiens

>NODE_4_length_491_cov_20.640110
GACCATCTAGCGACCTCCACCTTCCTGTGCGACTTGTTTGTCTCCCAGTCAAGCACCCTTGTGCCATTACACTCTGCGACCGGTTACCAATCGGCCTGAGGGTACCTT
TAGAAGCCTCCGTTACGCTTTTGGAGGCGACCACCCCAGTCAAACTACCCACCATACAGTGTCCTCGCAAAACGCGAGTTAGTATCCAAACATCAAAAGGGCCGTA
TTTCAACAACGACTCCACAAATACTGGCGTACCTGCTTCTAAGTCTCCGGCCTATCCTACACATCTGATGCCCAAATACAATGTAAAGCTATAGTAAAGGTTCACGG
GGTCTTTTCGTCCCGTTGCGGGTAATCGGCATCTTCACCGATACTACAATTTCACCGAGTTCGCGGTTGAGACAGTGCCCAGATCGTTACACCATTCGTGGAGGTCG
CTAGATGGTCAGATCGGAAGAGCGTCGTGTAGGGAAAGAGTGTGATTCTGCGTGTAGATCTCT

Accession	Coverage	Identity	Release_Date	Species	Molecule_type	Length	Geo_Location
OV195202.1	10	90	2021-12-15T00:00:00Z	Severe acute respiratory syndrome-related coronavirus	ssRNA(+)	30221	Germany:Europe/Germany/Baden-Wurttemberg
OV195277.1	10	88	2021-12-15T00:00:00Z	Severe acute respiratory syndrome-related coronavirus	ssRNA(+)	30090	Germany:Europe/Germany/Baden-Wurttemberg

OV195597.1	10	92	2021-12-15T00:00:00Z	Severe acute respiratory syndrome-related coronavirus	ssRNA(+)	30117	Germany:Europe/Germany/Baden-Wurttemberg
OV195847.1	6	100	2021-12-15T00:00:00Z	Severe acute respiratory syndrome-related coronavirus	ssRNA(+)	30308	Germany:Europe/Germany/Baden-Wurttemberg
OV195900.1	10	94	2021-12-15T00:00:00Z	Severe acute respiratory syndrome-related coronavirus	ssRNA(+)	30109	Germany:Europe/Germany/Baden-Wurttemberg
OV195924.1	10	89	2021-12-15T00:00:00Z	Severe acute respiratory syndrome-related coronavirus	ssRNA(+)	30333	Germany:Europe/Germany/Baden-Wurttemberg
OV196000.1	6	100	2021-12-15T00:00:00Z	Severe acute respiratory syndrome-related coronavirus	ssRNA(+)	30106	Germany:Europe/Germany/Baden-Wurttemberg
OV196022.1	10	90	2021-12-15T00:00:00Z	Severe acute respiratory syndrome-related coronavirus	ssRNA(+)	30562	Germany:Europe/Germany/Baden-Wurttemberg
OV196096.1	6	100	2021-12-15T00:00:00Z	Severe acute respiratory syndrome-related coronavirus	ssRNA(+)	29646	Germany:Europe/Germany/Baden-Wurttemberg
OV196177.1	10	90	2021-12-15T00:00:00Z	Severe acute respiratory syndrome-related coronavirus	ssRNA(+)	30003	Germany:Europe/Germany/Baden-Wurttemberg
OV196273.1	7	100	2021-12-15T00:00:00Z	Severe acute respiratory syndrome-related coronavirus	ssRNA(+)	30444	Germany:Europe/Germany/Baden-Wurttemberg
OV196275.1	10	92	2021-12-15T00:00:00Z	Severe acute respiratory syndrome-related coronavirus	ssRNA(+)	30423	Germany:Europe/Germany/Baden-Wurttemberg
OV196298.1	10	92	2021-12-15T00:00:00Z	Severe acute respiratory syndrome-related coronavirus	ssRNA(+)	30559	Germany:Europe/Germany/Baden-Wurttemberg
OV196467.1	9	91	2021-12-15T00:00:00Z	Severe acute respiratory syndrome-related coronavirus	ssRNA(+)	30053	Germany:Europe/Germany/Baden-Wurttemberg
OV196488.1	10	90	2021-12-15T00:00:00Z	Severe acute respiratory syndrome-related coronavirus	ssRNA(+)	30094	Germany:Europe/Germany/Baden-Wurttemberg
OV196492.1	10	89	2021-12-15T00:00:00Z	Severe acute respiratory syndrome-related coronavirus	ssRNA(+)	30243	Germany:Europe/Germany/Baden-Wurttemberg
OV196499.1	7	100	2021-12-15T00:00:00Z	Severe acute respiratory syndrome-related coronavirus	ssRNA(+)	30525	Germany:Europe/Germany/Baden-Wurttemberg
OV196584.1	6	100	2021-12-15T00:00:00Z	Severe acute respiratory syndrome-related coronavirus	ssRNA(+)	30066	Germany:Europe/Germany/Baden-Wurttemberg
OV196890.1	10	92	2021-12-15T00:00:00Z	Severe acute respiratory syndrome-related coronavirus	ssRNA(+)	30101	Germany:Europe/Germany/Baden-Wurttemberg
OV197136.1	10	90	2021-12-15T00:00:00Z	Severe acute respiratory syndrome-related coronavirus	ssRNA(+)	30102	Germany:Europe/Germany/Baden-Wurttemberg
OV197187.1	10	90	2021-12-15T00:00:00Z	Severe acute respiratory syndrome-related coronavirus	ssRNA(+)	30208	Germany:Europe/Germany/Baden-Wurttemberg

OV197189.1	10	90	2021-12-15T00:00:00Z	Severe acute respiratory syndrome-related coronavirus	ssRNA(+)	30094	Germany:Europe/Germany/Baden-Wurttemberg
OV197539.1	10	94	2021-12-15T00:00:00Z	Severe acute respiratory syndrome-related coronavirus	ssRNA(+)	30057	Germany:Europe/Germany/Baden-Wurttemberg
OV197600.1	10	90	2021-12-15T00:00:00Z	Severe acute respiratory syndrome-related coronavirus	ssRNA(+)	30105	Germany:Europe/Germany/Baden-Wurttemberg
OV016109.1	10	90	2021-11-23T00:00:00Z	Severe acute respiratory syndrome-related coronavirus	ssRNA(+)	30090	Germany:Europe/Germany/Baden-Wurttemberg
LC315647.2	7	97	2017-08-09T00:00:00Z	Betacoronavirus 1	ssRNA(+)	30755	Japan: Tokyo

Sample 5, *Rhinolophus hipposideros*, Female, Adygea Republic, rectal swab.

>NODE_2_length_1205_cov_11.209647
GTTTTTTTTTTTTTTTTCAAGCAGAAGACGGCATACGAGATCAGTTCCGGTGACGGAGTTCAGACGTGTGCTCTTCCGATCTGACCATCTAGCGACCTCCACAGTCCT
CGATCGATTAGTATTCGTCAGCTCCATGTGTCACCACACTTCCACCTCGAACCTATCTACCTCATCGTCTTTGAGGGATCTTACTTACTTGCGTAATGGGAAATCTCA
TCTTGAGGGGGGCTTCATGCTTAGATGCTTTCAGCACTTATCCCGTCCACACATAGCTACCCAGCGATGCCTTTGGCAAGACAACTGGTACACCAGCGGTGTGTCCA
TCCCGGTCCTCTCGTACTAAGGACAGCTCCTCTCAAATTTCCTACGCCCACGACGGATAGGGACCGAACTGTCTCACGACGTTCTGAACCCAGCTCGCGTACCGCTT
TAATGGGCGAACAGCCCAACCCTTGGGACCGACTACAGCCCCAGGATGCGATGAGCCGACATCGAGGTGCCAAACCTCCCCGTCGATGTGGACTCTTGGGGGAGA
TAAGCCTGTTATCCCCGGGGTAGCTTTTATCCGTTGAGCGATGGCCCTTCCATGCGGAACCACCGGATCACTAAGCCCGTCTTTCGACCCTGCTCGACTTGTAGGTCT
CGCAGTCAAGCTCCCTTGTGCCTTTACACTCTACGAATGATTTCCAACCATTCTGAGGGAACCTTTGGGCGCCTCCGTTACCTTTTAGGAGGCGACCGCCCCAGTCA
AACTGTCCGCCTGACACTGTCTCCTGCCCCGCTAAGGGGCATGGGTTAGAATTTCAATACAACCAGGGTAGTATCCCACCGACGCCTCCTTCGAAGCTGGCGCTCCG
AGATCTCTGGCTCCTACCTATCCTGTACAAGTTGTACCAAAATTCAATATCAGGCTACAGTAAAGCTCCACGGGGTCTTTCCGTCCTGTCGCGGGTAACCTGCATCTT
CACAGGTACTATAATTTCACCGAGTCTCTCGTTGAGACAGTGCCCAGATCGTTACGCCTTTCGTGGAGGTCGCTAGATGGTCAAGAGCACACGTCTGAACTCCAGTC
ACCGGAACTGATCTCGTATGCCGTCTTCTGCTTGAAAAAAAAAAAAACACCCACTACTGATATGAGATAACGTACTGAGTACTCCTTAGCACATCAATCATCTAACA
AACAAAAAATAATCTAAATACAAACAC

Accession	Coverage	Identity	Release_Date	Species	Molecule_type	Length	Geo_Location	Host
MZ680132.1	4	100	2022-03-10T00:00:00Z	Narnaviridae sp.	ssRNA(+)	3644	China: Hubei	
MZ679269.1	9	85	2022-02-23T00:00:00Z	Sichuan sapelo-like virus 1	ssRNA(+)	4686	China: Hainan	
OV377758.1	6	91	2022-01-09T00:00:00Z	Severe acute respiratory syndrome-related coronavirus	ssRNA(+)	30273	Germany:Europe/Germany/Baden-Wurttemberg	Homo sapiens
BK059322.1	2	97	2021-12-18T00:00:00Z	Arabis mosaic virus	ssRNA(+)	7408		Narcissus pseudonarcissus
OV195107.1	6	91	2021-12-15T00:00:00Z	Severe acute respiratory syndrome-related coronavirus	ssRNA(+)	30103	Germany:Europe/Germany/Baden-Wurttemberg	Homo sapiens

OV195202.1	6	89	2021-12-15T00:00:00Z	Severe acute respiratory syndrome-related coronavirus	ssRNA(+)	30221	Germany:Europe/Germany/Baden-Wurttemberg	Homo sapiens
OV195277.1	6	91	2021-12-15T00:00:00Z	Severe acute respiratory syndrome-related coronavirus	ssRNA(+)	30090	Germany:Europe/Germany/Baden-Wurttemberg	Homo sapiens
OV195597.1	6	94	2021-12-15T00:00:00Z	Severe acute respiratory syndrome-related coronavirus	ssRNA(+)	30117	Germany:Europe/Germany/Baden-Wurttemberg	Homo sapiens
OV195847.1	12	91	2021-12-15T00:00:00Z	Severe acute respiratory syndrome-related coronavirus	ssRNA(+)	30308	Germany:Europe/Germany/Baden-Wurttemberg	Homo sapiens
OV195900.1	5	91	2021-12-15T00:00:00Z	Severe acute respiratory syndrome-related coronavirus	ssRNA(+)	30109	Germany:Europe/Germany/Baden-Wurttemberg	Homo sapiens
OV195924.1	6	93	2021-12-15T00:00:00Z	Severe acute respiratory syndrome-related coronavirus	ssRNA(+)	30333	Germany:Europe/Germany/Baden-Wurttemberg	Homo sapiens
OV196000.1	6	91	2021-12-15T00:00:00Z	Severe acute respiratory syndrome-related coronavirus	ssRNA(+)	30106	Germany:Europe/Germany/Baden-Wurttemberg	Homo sapiens
OV196009.1	6	94	2021-12-15T00:00:00Z	Severe acute respiratory syndrome-related coronavirus	ssRNA(+)	30101	Germany:Europe/Germany/Baden-Wurttemberg	Homo sapiens
OV196022.1	12	92	2021-12-15T00:00:00Z	Severe acute respiratory syndrome-related coronavirus	ssRNA(+)	30562	Germany:Europe/Germany/Baden-Wurttemberg	Homo sapiens
OV196026.1	12	92	2021-12-15T00:00:00Z	Severe acute respiratory syndrome-related coronavirus	ssRNA(+)	30121	Germany:Europe/Germany/Baden-Wurttemberg	Homo sapiens
OV196099.1	6	91	2021-12-15T00:00:00Z	Severe acute respiratory syndrome-related coronavirus	ssRNA(+)	30100	Germany:Europe/Germany/Baden-Wurttemberg	Homo sapiens
OV196107.1	6	93	2021-12-15T00:00:00Z	Severe acute respiratory syndrome-related coronavirus	ssRNA(+)	30511	Germany:Europe/Germany/Baden-Wurttemberg	Homo sapiens
OV196177.1	6	92	2021-12-15T00:00:00Z	Severe acute respiratory syndrome-related coronavirus	ssRNA(+)	30003	Germany:Europe/Germany/Baden-Wurttemberg	Homo sapiens
OV196273.1	12	95	2021-12-15T00:00:00Z	Severe acute respiratory syndrome-related coronavirus	ssRNA(+)	30444	Germany:Europe/Germany/Baden-Wurttemberg	Homo sapiens
OV196275.1	6	91	2021-12-15T00:00:00Z	Severe acute respiratory syndrome-related coronavirus	ssRNA(+)	30423	Germany:Europe/Germany/Baden-Wurttemberg	Homo sapiens
OV196298.1	12	91	2021-12-15T00:00:00Z	Severe acute respiratory syndrome-related coronavirus	ssRNA(+)	30559	Germany:Europe/Germany/Baden-Wurttemberg	Homo sapiens
OV196467.1	5	94	2021-12-15T00:00:00Z	Severe acute respiratory syndrome-related coronavirus	ssRNA(+)	30053	Germany:Europe/Germany/Baden-Wurttemberg	Homo sapiens
OV196488.1	12	92	2021-12-15T00:00:00Z	Severe acute respiratory syndrome-related coronavirus	ssRNA(+)	30094	Germany:Europe/Germany/Baden-Wurttemberg	Homo sapiens
OV196492.1	12	93	2021-12-15T00:00:00Z	Severe acute respiratory syndrome-related coronavirus	ssRNA(+)	30243	Germany:Europe/Germany/Baden-Wurttemberg	Homo sapiens

OV196584.1	6	91	2021-12-15T00:00:00Z	Severe acute respiratory syndrome-related coronavirus	ssRNA(+)	30066	Germany:Europe/Germany/Baden-Wurttemberg	Homo sapiens
OV196626.1	6	90	2021-12-15T00:00:00Z	Severe acute respiratory syndrome-related coronavirus	ssRNA(+)	30095	Germany:Europe/Germany/Baden-Wurttemberg	Homo sapiens
OV196890.1	6	91	2021-12-15T00:00:00Z	Severe acute respiratory syndrome-related coronavirus	ssRNA(+)	30101	Germany:Europe/Germany/Baden-Wurttemberg	Homo sapiens
OV197136.1	6	91	2021-12-15T00:00:00Z	Severe acute respiratory syndrome-related coronavirus	ssRNA(+)	30102	Germany:Europe/Germany/Baden-Wurttemberg	Homo sapiens
OV197187.1	6	91	2021-12-15T00:00:00Z	Severe acute respiratory syndrome-related coronavirus	ssRNA(+)	30208	Germany:Europe/Germany/Baden-Wurttemberg	Homo sapiens
OV197189.1	6	91	2021-12-15T00:00:00Z	Severe acute respiratory syndrome-related coronavirus	ssRNA(+)	30094	Germany:Europe/Germany/Baden-Wurttemberg	Homo sapiens
OV197487.1	5	89	2021-12-15T00:00:00Z	Severe acute respiratory syndrome-related coronavirus	ssRNA(+)	30104	Germany:Europe/Germany/Baden-Wurttemberg	Homo sapiens
OV197539.1	12	96	2021-12-15T00:00:00Z	Severe acute respiratory syndrome-related coronavirus	ssRNA(+)	30057	Germany:Europe/Germany/Baden-Wurttemberg	Homo sapiens
OV197600.1	6	92	2021-12-15T00:00:00Z	Severe acute respiratory syndrome-related coronavirus	ssRNA(+)	30105	Germany:Europe/Germany/Baden-Wurttemberg	Homo sapiens
OV015804.1	6	91	2021-11-23T00:00:00Z	Severe acute respiratory syndrome-related coronavirus	ssRNA(+)	29996	Germany:Europe/Germany/Baden-Wurttemberg	Homo sapiens
OV016108.1	12	90	2021-11-23T00:00:00Z	Severe acute respiratory syndrome-related coronavirus	ssRNA(+)	30390	Germany:Europe/Germany/Baden-Wurttemberg	Homo sapiens
OV016109.1	6	91	2021-11-23T00:00:00Z	Severe acute respiratory syndrome-related coronavirus	ssRNA(+)	30090	Germany:Europe/Germany/Baden-Wurttemberg	Homo sapiens
LP633679.1	6	96	2016-02-19T00:00:00Z	Qubevirus durum	ssRNA(+)	712		Escherichia

>NODE_5_length_465_cov_1.026627
TTCTCACTTTCGTCTGATTTGTTCTTGTCTGTTCTTTACTTGTGGTATACGCTATGATTGGTTGTGTGGTTCTTCGTTGGTTCTTTGTTTTTTTTTTTTCAAGCAGAAGAC
GGCATACGAGATCAGTTCCGGTGACTGGAGTTCAGACGTGTGCTCTCCGATCTGACCATCTAGCGACCTCCACTTTCCGGCTTCATGTAGGCGAGTTGCAGCCTACA
ATCCGAACTGAGAACGACTTTATCGGATTAGCTCCCTCTCGCGAGTTGGCAACCGTTTGTATCGTGGAGGTCGCTAGATGGTCGATCGGAAGAGCGTCGTGTAGGG
AAAGAGGGTTCGTAGTTGTGTAGATCTCGGTGGTCGCCGGAGCGTTATAAAAAACAGAATAGACATAGAATAGACATGCAGAGTATAGAGAAGTGCAAAAATACG
ACTAAAGTAGGAGGTGGTATCTAAGCATGCGTGGGT

Accession	Coverage	Identity	Release_Date	Species	Molecule_type	Length	Geo_Location	Host
OV195202.1	16	87	2021-12-15T00:00:00Z	Severe acute respiratory syndrome-related coronavirus	ssRNA(+)	30221	Germany:Europe/Germany/Baden-Wurttemberg	Homo sapiens

OV195277.1	16	88	2021-12-15T00:00:00Z	Severe acute respiratory syndrome-related coronavirus	ssRNA(+)	30090	Germany:Europe/Germany/Baden-Wurttemberg	Homo sapiens
OV195597.1	12	89	2021-12-15T00:00:00Z	Severe acute respiratory syndrome-related coronavirus	ssRNA(+)	30117	Germany:Europe/Germany/Baden-Wurttemberg	Homo sapiens
OV195847.1	6	100	2021-12-15T00:00:00Z	Severe acute respiratory syndrome-related coronavirus	ssRNA(+)	30308	Germany:Europe/Germany/Baden-Wurttemberg	Homo sapiens
OV195900.1	16	87	2021-12-15T00:00:00Z	Severe acute respiratory syndrome-related coronavirus	ssRNA(+)	30109	Germany:Europe/Germany/Baden-Wurttemberg	Homo sapiens
OV195924.1	16	89	2021-12-15T00:00:00Z	Severe acute respiratory syndrome-related coronavirus	ssRNA(+)	30333	Germany:Europe/Germany/Baden-Wurttemberg	Homo sapiens
OV196000.1	6	100	2021-12-15T00:00:00Z	Severe acute respiratory syndrome-related coronavirus	ssRNA(+)	30106	Germany:Europe/Germany/Baden-Wurttemberg	Homo sapiens
OV196022.1	10	88	2021-12-15T00:00:00Z	Severe acute respiratory syndrome-related coronavirus	ssRNA(+)	30562	Germany:Europe/Germany/Baden-Wurttemberg	Homo sapiens
OV196096.1	12	89	2021-12-15T00:00:00Z	Severe acute respiratory syndrome-related coronavirus	ssRNA(+)	29646	Germany:Europe/Germany/Baden-Wurttemberg	Homo sapiens
OV196177.1	12	90	2021-12-15T00:00:00Z	Severe acute respiratory syndrome-related coronavirus	ssRNA(+)	30003	Germany:Europe/Germany/Baden-Wurttemberg	Homo sapiens
OV196273.1	33	95	2021-12-15T00:00:00Z	Severe acute respiratory syndrome-related coronavirus	ssRNA(+)	30444	Germany:Europe/Germany/Baden-Wurttemberg	Homo sapiens
OV196275.1	16	87	2021-12-15T00:00:00Z	Severe acute respiratory syndrome-related coronavirus	ssRNA(+)	30423	Germany:Europe/Germany/Baden-Wurttemberg	Homo sapiens
OV196298.1	16	87	2021-12-15T00:00:00Z	Severe acute respiratory syndrome-related coronavirus	ssRNA(+)	30559	Germany:Europe/Germany/Baden-Wurttemberg	Homo sapiens
OV196467.1	6	100	2021-12-15T00:00:00Z	Severe acute respiratory syndrome-related coronavirus	ssRNA(+)	30053	Germany:Europe/Germany/Baden-Wurttemberg	Homo sapiens
OV196488.1	33	92	2021-12-15T00:00:00Z	Severe acute respiratory syndrome-related coronavirus	ssRNA(+)	30094	Germany:Europe/Germany/Baden-Wurttemberg	Homo sapiens
OV196492.1	11	88	2021-12-15T00:00:00Z	Severe acute respiratory syndrome-related coronavirus	ssRNA(+)	30243	Germany:Europe/Germany/Baden-Wurttemberg	Homo sapiens
OV196499.1	16	86	2021-12-15T00:00:00Z	Severe acute respiratory syndrome-related coronavirus	ssRNA(+)	30525	Germany:Europe/Germany/Baden-Wurttemberg	Homo sapiens
OV196584.1	9	93	2021-12-15T00:00:00Z	Severe acute respiratory syndrome-related coronavirus	ssRNA(+)	30066	Germany:Europe/Germany/Baden-Wurttemberg	Homo sapiens
OV196890.1	12	88	2021-12-15T00:00:00Z	Severe acute respiratory syndrome-related coronavirus	ssRNA(+)	30101	Germany:Europe/Germany/Baden-Wurttemberg	Homo sapiens
OV197136.1	16	85	2021-12-15T00:00:00Z	Severe acute respiratory syndrome-related coronavirus	ssRNA(+)	30102	Germany:Europe/Germany/Baden-Wurttemberg	Homo sapiens

OV197187.1	13	90	2021-12-15T00:00:00Z	Severe acute respiratory syndrome-related coronavirus	ssRNA(+)	30208	Germany:Europe/Germany/Baden-Wurttemberg	Homo sapiens
OV197189.1	16	85	2021-12-15T00:00:00Z	Severe acute respiratory syndrome-related coronavirus	ssRNA(+)	30094	Germany:Europe/Germany/Baden-Wurttemberg	Homo sapiens
OV197539.1	33	96	2021-12-15T00:00:00Z	Severe acute respiratory syndrome-related coronavirus	ssRNA(+)	30057	Germany:Europe/Germany/Baden-Wurttemberg	Homo sapiens
OV197600.1	16	89	2021-12-15T00:00:00Z	Severe acute respiratory syndrome-related coronavirus	ssRNA(+)	30105	Germany:Europe/Germany/Baden-Wurttemberg	Homo sapiens
OV016109.1	16	86	2021-11-23T00:00:00Z	Severe acute respiratory syndrome-related coronavirus	ssRNA(+)	30090	Germany:Europe/Germany/Baden-Wurttemberg	Homo sapiens
LC315647.2	6	100	2017-08-09T00:00:00Z	Betacoronavirus 1	ssRNA(+)	30755	Japan: Tokyo	Homo sapiens
